# Preparation and Characterization of Fenofibrate Microparticles with Surface-Active Additives: Application of a Supercritical Fluid-Assisted Spray-Drying Process

**DOI:** 10.3390/pharmaceutics13122061

**Published:** 2021-12-02

**Authors:** Jeong-Soo Kim, Heejun Park, Eun-Sol Ha, Kyu-Tae Kang, Min-Soo Kim, Sung-Joo Hwang

**Affiliations:** 1 Dong-A ST Co., Ltd., Giheung-gu, Yongin 446-905, Korea; js_kim@donga.co.kr; 2 College of Pharmacy, Duksung Women’s University, 33, Samyangro 144-gil, Dobong-gu, Seoul 01369, Korea; heejunpark@duksung.ac.kr (H.P.); ktkang@duksung.ac.kr (K.-T.K.); 3 College of Pharmacy, Pusan National University, 63 Busandaehak-ro, Geumjeong-gu, Busan 46241, Korea; edel@pusan.ac.kr; 4 Yonsei Institute of Pharmaceutical Sciences, College of Pharmacy, Yonsei University, 85 Songdogwahak-ro, Yeonsu-gu, Incheon 21983, Korea

**Keywords:** supercritical fluid assisted spray-drying (SA-SD), fenofibrate, surface-active additive, spray-drying (SD), microparticle, biopharmaceutical performance

## Abstract

In this study, supercritical fluid-assisted spray-drying (SA-SD) was applied to achieve the micronization of fenofibrate particles possessing surface-active additives, such as d-α-tocopheryl polyethylene glycol 1000 succinate (TPGS), sucrose mono palmitate (Sucroester 15), and polyoxyethylene 52 stearate (Myrj 52), to improve the pharmacokinetic and pharmacodynamic properties of fenofibrate. For comparison, the same formulation was prepared using a spray-drying (SD) process, and then both methods were compared. The SA-SD process resulted in a significantly smaller mean particle size (approximately 2 μm) compared to that of unprocessed fenofibrate (approximately 20 μm) and SD-processed particles (approximately 40 μm). There was no significant difference in the effect on the particle size reduction among the selected surface-active additives. The microcomposite particles prepared with surface-active additives using SA-SD exhibited remarkable enhancement in their dissolution rate due to the synergistic effect of comparably moderate wettability improvement and significant particle size reduction. In contrast, the SD samples with the surface-active additives exhibited a decrease in dissolution rate compared to that of the unprocessed fenofibrate due to the absence of particle size reduction, although wettability was greatly improved. The results of zeta potential and XPS analyses indicated that the surface-active additive coverage on the surface layer of the SD-processed particles with a better wettability was higher than that of the SA-SD-processed composite particles. Additionally, after rapid depletion of hydrophilic additives that were excessively distributed on the surfaces of SD-processed particles, the creation of a surface layer rich in poorly water-soluble fenofibrate resulted in a decrease in the dissolution rate. In contrast, the surface-active molecules were dispersed homogeneously throughout the particle matrix in the SA-SD-processed microparticles. Furthermore, improved pharmacokinetic and pharmacodynamic characteristics were observed for the SA-SD-processed fenofibrate microparticles compared to those for the SD-processed fenofibrate particles. Therefore, the SA-SD process incorporating surface-active additives can efficiently micronize poorly water-soluble drugs and optimize their physicochemical and biopharmaceutical characteristics.

## 1. Introduction

Biopharmaceutics Classification System (BCS) class II drugs must overcome challenges during the pharmaceutical development process that include determining a means to enable sufficient bioavailability due to their low solubility. Drugs that exhibit poor aqueous solubility do not dissolve rapidly and, thus, may not be adequately absorbed through the oral route of administration. Therefore, major efforts have been made in the pharmaceutical industry to improve the bioavailability and/or the onset of action of these drugs by focusing on increasing the dissolution rate of poorly water-soluble drugs via the reduction of particle size (i.e., micronization) through the use of hydrophilic surface-active materials [1,2,3,4,5,6,7,8]. In response to these needs, microparticle preparation technologies using supercritical fluids (SCF), particularly carbon dioxide, have been applied to improve the physicochemical properties of drug particles via fine control of particle precipitation. The advantages of particle formation processes using supercritical carbon dioxide (SC-CO_2_) include rapid and efficient mass transfer and higher solvent power compared to these characteristics using conventional solvents [9,10]. Additionally, these SCF properties can be finely adjusted by varying several process parameters, such as pressure and temperature. In general, particle formation techniques using SC-CO_2_ are classified into four groups based on the solvating behavior of SCF, including (i) rapid expansion of supercritical solution (RESS) process using SCF as a solvent [11,12,13,14,15,16,17,18,19,20,21], (ii) supercritical anti-solvent (SAS) processes using SCF as an antisolvent [22,23,24,25,26], (iii) particles from a gas saturated solution (PGSS) process using as a solute [27,28,29,30,31], and (iv) several processes using SCF as an atomizing agent, such as carbon dioxide-assisted nebulization with a bubble fryer^®^ (CAN-BD) [32], supercritical fluid-assisted atomization (SAA) [33], and supercritical fluid-assisted spray-drying (SASD) [34,35]. These SCF technologies have been introduced as promising particle engineering techniques for the control of particle size from micro- to nano-meter dimensions with a narrow particle size distribution (PSD) [36,37,38,39,40,41,42,43,44,45,46,47,48].

In particular, several processes incorporating the use of SCF have recently been used to allow atomizing agents to nebulize organic solutions containing substrates, and these have received much attention. These processes permit the treatment of any compound regardless of the compound’s solubility in SC-CO_2_. The substrate is dissolved into an organic or an aqueous solution, which is mixed under pressure with the SCF, forming a mixed fluid. Then, the mixed fluid flows through a restrictor and is rapidly expanded through the suitable device to form an aerosol. The aerosol droplets are dried to form fine particles [49,50,51]. These processes provide very efficient and versatile methods for particle formation in the context of drug delivery systems. It is clear that the SAA and CAN-BD processes overcome a number of the drawbacks that are inherent to conventional processes that use SCF. Unfortunately, several problems, such as the recovery of produced nanoparticles, the requirement of high temperatures for evaporating liquid solvents, and complications related to the application of practical drug production related to GMP compliance, still exist.

Based on the principle regarding SAA and CAN-BD processes, a group led by Hwang developed a novel SA-SD process as a valid alternative to the conventional SD process and the SAS process for the preparation of nanoparticles [34,35]. In the SA-SD process, SC-CO_2_ acts both as a co-solvent that is miscible with the drug solution to be treated and as an atomizing agent that can atomize the drug solution in a fine droplet. Moreover, organic and inorganic solvents can be used for fine particle formation of both hydrophilic and hydrophobic drugs [49,51,52,53,54,55,56,57]. 

In the present study, fenofibrate was chosen as the model compound due to its practical insolubility in water. The SA-SD process was applied to achieve the micronization of fenofibrate particles possessing several surface-active additives, such as α-tocopheryl polyethelyene glycol 1000 succinate (TPGS), sucrose monopalmitate (Sucroester 15), and polyoxyethylene 40 stearate (Myrj 52), to enhance the wettability and dissolution rate and, thus, improve the pharmacokinetic (PK) and pharmacodynamic (PD) effects of fenofibrate. Among the various SA-SD process parameters, critical factors, such as concentration of drug solution, CO_2_ injection rate, and contents of surface-active additives [51], that can show a dramatic effect on the performance of the SA-SD processed particles were evaluated through experimental design using a three-factor, three-level Box–Behnken design (BBD) with three replicates at the center point to build response surface models, and the optimized process parameters and the optimized formula were, thus, determined. Finally, the SA-SD process was compared to the spray-drying (SD) process in terms of the physicochemical and biological performance of the composite particles produced by two different processes. Physicochemical characterizations, including particle size analysis, contact angle measurement, zeta potential measurement, SEM, DSC, PXRD, FT-IR, contact angle measurement, and dissolution tests, were also performed to evaluate the effect of the addition of hydrophilic additives on the in vitro performance and morphology of the SA-SD-processed fenofibrate particles. Additionally, the effect of the dissolution rate enhancement on the pharmacokinetic and pharmacodynamic performance of fenofibrate was studied in Sprague–Dawley rats.

## 2. Materials and Methods

### 2.1. Materials

Fenofibrate (MW 360.84) and Myrj 52 (MW 2046.58) were supplied from Sigma (St. Louis, MO, USA). TPGS (MW 1513) was obtained from Eastman Chemical Co. (Kingsport, TN, USA). Sucroester 15 (MW 580.71) was kindly supplied by Gattefossè (Saint-Priest, France). The chemical structures of fenofibrate and additives used are presented in Figure S1 (Supplementary Material). Carbon dioxide (CO_2_, purity 99.9%) was supplied from Hanmi Gas Co., Ltd. (Daejeon, Korea). Sodium lauryl sulfate (extra-pure grade) was obtained from Duksan Pure Chemicals (Ansan, Korea). Ethanol (purity 99.85%) was supplied from Hayman Ltd. (Essex, UK). All other solvents were of HPLC grade.

### 2.2. Preparation of Fenofibrate Microparticles 

#### 2.2.1. SA-SD Process

**Figure 1:**
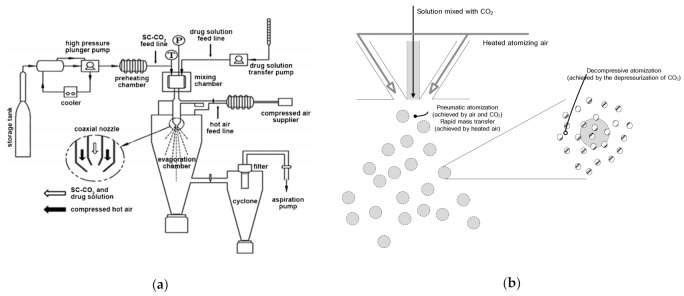
Schematic diagram of (**a**) the apparatus (reprinted from Reference [25] with permission, Elsevier 2015) and (**b**) the atomization mechanism the SA-SD process.

Figure 1a,b, respectively, depict the SA-SD apparatus and its atomization mechanism that was used to perform all the experiments in this study. The SA-SD apparatus consists of four feed lines used to deliver CO_2_, the drug solution, atomizing air, drying air, and two process vessels, including a mixing chamber (approximate internal volume 30 cm^3^) and a precipitator (approximate internal volume 6000 cm^3^). Liquid CO_2_ and the drug/additive solution were introduced at constant feed rates into the mixing chamber using two high pressure pumps that included a Suflux^®^ plunger type metering pump (Ilshin Autoclave Co., Daejeon, Korea) and a high-pressure liquid pump (model 307, Gilson, Middleton, WI, USA) for CO_2_ and the drug/additive solution, respectively. The mixing chamber is a high-pressure vessel loaded with glass beads (diameter 1.5 mm) for vigorous mixing of CO_2_ and the drug solution. The mixture of CO_2_ and the drug/additive solution was transported from the mixing chamber to the coaxial nozzle and then sprayed into the precipitator with heated air for rapid mass transfer. In the precipitator, an additional heated air flow was also delivered for rapid evaporation of ethanol that was used as a solvent. The pressure of the precipitator was maintained close to atmospheric conditions using an aspiration pump (aspiration flow 400 L/min, Woosung Vacuum, Jeju, Korea) during the particle formation process. All particle formation processes were performed at the optimal pressure and temperature to achieve complete miscibility of CO_2_ + ethanol + fenofibrate + additive by adjusting the valve located in the coaxial nozzle. After the particle formation process, the precipitated fenofibrate microparticles were collected from the baffle dust collector that was designed to allow the gas stream to make a sudden change of direction and from the wire mesh filter located at the bottom of the precipitator.

#### 2.2.2. Box–Behnken Design (BBD)

Response surface methodology was combined with Box–Behnken design (BBD) to determine the effect of the additives on the SA-SD process performance and the two-factor interaction between the CO_2_ injection rate and drug solution concentration, and this approach was also used to investigate the effect of the additives on the SA-SD processed particles. A three-factor and three-level BBD with three replicates of the center point was selected to generate the response surface models. The three factors included the drug/additive solution concentration (X1), the CO_2_ injection rate (X2), and the content of additive (X3), and the responses were the mean particle size, the SPAN value, and the dissolution efficiency of fenofibrate at 30 min (DE_30_). The DE calculation method is mentioned in below Section 2.3.8. The experimental runs with independent variables, including the drug/additive solution concentration, CO_2_ injection rate, and additive content, are presented in Tables S1–S3 (Supplementary Material) for Sucroester 15, TPGS, and Myrj 52, respectively. The design was constructed using Design-Expert software (version 7.0, Stat-Ease, Inc., Minneapolis, MN, USA).

#### 2.2.3. Preparation of Fenofibrate Microcomposite Particles under Optimized Conditions

Briefly, solutions of fenofibrate/Sucroester 15, fenofibrate/TPGS, and fenofibrate/Myrj 52 in ethanol were prepared. Then, a fenofibrate/additive solution (20 mg/mL) and supercritical CO_2_ were co-injected into the mixing chamber that was filled with supercritical CO_2_ (12 MPa, 40 °C) at 10 mL/min and 35 g/min, respectively. Other process conditions were as follows: drying air inlet temperature, 65 °C; outlet temperature, 35~40 °C; atomization air pressure, 0.3 MPa; aspiration velocity, 0.40 m^3^/min. During the SA-SD process, the pressure of the mixing chamber was constantly controlled using a needle valve located on the coaxial nozzle. After the particle formation process, fenofibrate composite particles were collected from the baffle dust collector that was designed to allow the gas stream to make a sudden change in direction and from the wire mesh filter that was located at the bottom of the precipitator. The formulations of the SA-SD-processed fenofibrate microparticles with surface-active excipients are summarized in Table 1.

#### 2.2.4. Conventional Spray-Drying (SD) Process

Fenofibrate and each additive were dissolved in ethanol to obtain a clear solution. The spray drying process was performed using the SA-SD apparatus without CO_2_ flow at conditions that included: 20 mg/mL of drug solution concentration; 65 °C drying air inlet temperature; 35~40 °C outlet temperature; 10 mL/min for drug solution feeding speed; 0.3 MPa atomization air pressure; 0.40 m^3^/min aspiration velocity. The formulations of the spray-dried fenofibrate microparticles are presented in Table 1.

### 2.3. Physicochemical Characterization of Fenofibrate Microparticles

#### 2.3.1. Particle Size Analysis

The analysis examining prepared particle size was conducted using a Microtrac^®^ X-100 (Honeywell, Montgomeryville, PA, USA) based on the laser diffraction method. The Microtrac^®^ X-100 system possesses a detection range of 0.021–704 μm. Fenofibrate particles were dispersed in distilled water and sonicated prior to measurement to achieve complete dispersion.

#### 2.3.2. Powder X-ray Diffraction (PXRD)

The powder X-ray diffraction patterns of the samples were obtained using a Rigaku D/Max-2200 Ultima/PC powder X-ray diffraction system (Rigaku, Tokyo, Japan) with Ni-filtered Cu-Kα radiation. The 2θ scan range was 4–60° with a step size of 0.02° and a scan speed of 4° min^−1^.

#### 2.3.3. Differential Scanning Calorimetry (DSC)

Samples in the range of 2–5 mg of fenofibrate particles were added to crimp-sealed aluminum pans and measured on a Sinco S-650 DSC (Sinco, Seoul, Korea). The samples were heated at a rate of 5 °C/min from 30 °C to 100 °C. The samples were purged with nitrogen gas at 20 mL/min. The DSC was calibrated using an indium standard.

#### 2.3.4. Scanning Electron Microscopy (SEM)

SEM images of fenofibrate microparticles were obtained using a field-emission scanning electron microscope (JSM-7000F, JEOL Ltd., Tokyo, Japan). Fenofibrate particles were placed on aluminum stubs using double-adhesive carbon tape. Then, the particles were coated with fold/palladium using a FineCoat Sputter (JFC-1100, Jeol, Ltd., software (Akishima, Tokyo, Japan)).

#### 2.3.5. X-ray Photoelectron Spectroscopy (XPS)

XPS analysis was performed using a Thermo Multilab 2000 photoelectron spectrometer (Thermo Fisher Scientific, Waltham, MA, USA) to analyze the solid surfaces of SA-SD-processed fenofibrate microcomposite particles with surface-active additives [58]. The electrons emitted from the sample originate from the near-surface region of most solids (analysis depth: 10 nm). To avoid further scattering, the analysis must be performed in an ultra-high vacuum of 10^−8^ Torr. An Al Kα X-ray source was used in this instrument. The applied take-off angle of the photoelectrons for detection was perpendicular to the sample holder. For the analysis, it was assumed that all components existed in patches, that the patches were thicker than the analysis depth, and that the data for the atomic surface composition were converted into molecular surface composition based on the assumption that the surface composition is a linear combination of the different molecular species. The area analyzed consisted of a region <1 mm^2^, and three measurements were repeated three times for each sample.

#### 2.3.6. Zeta Potential Measurement

The zeta potential of SA-SD-processed fenofibrate particles was measured using an electrophoretic light scattering spectrophotometer (ELS-8000, Otsuka Electronics Co., Osaka, Japan). The powder samples were homogeneously dispersed in water and used for zeta potential analysis.

#### 2.3.7. Contact Angle Measurement

The contact angle measurements for the SA-SD-processed composite particles were performed using a Phoenix 300 contact angle analyzer (Surface Electro-Optics, Seoul, Korea) with a capture interval of 20 ms. The distilled water contained in a syringe impacted the pellets, and this followed by image capture and analysis. To form a pellet, an appropriate amount of solid sample was weighed and compacted using a Perkin Elmer hydraulic press (ATSFAAR, PerkinElmer, Inc., Waltham, MA, USA) at 5 t force for 10 s. As the contact angle decreased with time due to the spreading and absorption phenomena, the equilibrium contact angle was determined by extrapolating the fitted linear function at t = 0 [59]. The equilibrium contact angle measurements were performed at c to guarantee reproducibility.

#### 2.3.8. Dissolution Test

Dissolution tests of fenofibrate particles (equivalent to 160 mg as fenofibrate) were performed according to the USP XXVIII Type II (paddle method) using a VK7000 dissolution apparatus (VK7000, Vankel, Edison, NJ, USA). The speed of paddle rotation was 75 rpm, and 1000 mL of aqueous solution containing 0.025 M sodium lauryl sulfate was used as the dissolution medium. Samples (2 mL) were collected at predetermined sampling time points and then filtered using a 0.45 μm PTFE syringe filter. The concentration of the dissolved drug was measured by HPLC analysis. The HPLC system consisted of a pump (LC-10AD, Shimadzu, Kyoto, Japan) and a UV detector (SPD-10A, Shimadzu, Kyoto, Japan). The column used was Lichrospher^®^ RP 18 (4.6 × 50 mm, 5 μm, Merck, Darmstadt, Germany). The composition of the mobile phase was a mixture of water and acetonitrile (30:70) at pH 2.5. The flow rate and UV-detection wavelength condition were 1.2 mL/min and 286 nm, respectively. This HPLC analysis method was adopted from the assay method of fenofibrate tablet monograph in the United States Pharmacopeia/National Formulary (USP/NF) [60,61]. Dissolution efficiency (DE_t_) was calculated from the area under the dissolution curve at time t (measured using the trapezoidal rule) and expressed as the percentage of the area of the rectangle described by 100% dissolution at the same time [62]. All tests were performed in triplicate.

### 2.4. In Vivo Studies Using Sprague–Dawley Rats

Animal experiments were performed according to the guidelines for the care and use of laboratory animals. The animal experimental protocols (Approval Code: I-1708186) were approved by the Institutional Review Board of the nonclinical contract research organization KPC laboratory (approval date: 29 August 2017).

#### 2.4.1. Pharmacokinetic (PK) Study

Male Sprague–Dawley rats (6–7 weeks old, 180–200 g) were obtained from Samtaco Bio Korea, Inc. (Osan, Korea). The rats were housed in a cage and maintained on a 12-h light/dark cycle at room temperature (25 °C) and a relative humidity of 55 ± 10%. General and environmental conditions were strictly monitored. All rats were permitted free access to tap water and a pelleted diet during maintenance; however, they were deprived of food for 24 h prior to drug administration. Food intake was permitted again at 4 h after dosing. The rats were divided into seven groups consisting of seven animals each. SD-and SA-SD-processed powder samples were freshly prepared and used for animal studies. One milliliter of suspensions containing fenofibrate powder samples (equivalent to 50 mg/kg body weight as fenofibrate) in water with 0.2% *w/v* methylcellulose was administered to each group via oral administration using oral zonde. Blood samples were collected from the tail vein at predetermined time points that included pre-dose and 20, 40, 60, 90, 120, 180, 240, 360, 480, and 720 min post-dosing. The collected blood samples were stored in an ice bath at approximately 4 °C and then centrifuged at 4 °C and 10,000 rpm for 10 min. After centrifugation, serum was collected and stored in individual plastic tubes at −20 °C until analysis.

For the preparation of the HPLC analysis sample solution, 200 μL of each serum sample was transferred into a plastic tube and then spiked with 20 μL of internal standard solution (clofibric acid, 2000 μg/mL), and this was followed by the addition of 200 μL 1 M HCl. The mixture was mixed for 30 s using a vortexer, and 1 mL of n-hexane/ethyl acetate (9:1 *v*/*v*) mixture solution was then added and mixed for 5 min. After centrifugation at 13,000 rpm for 10 min, the collected clear supernatants were transferred to a tube and then heated (at 40 °C) to evaporate for drying under a nitrogen flow. After complete evaporation, the residue was reconstituted in 1 mL of solvent (distilled water/acetonitrile =7:3). To obtain the supernatant, the samples were centrifuged at 3000 rpm for 5 min. The clear 50 μL aliquot collected from the supernatant was injected into the HPLC system for the analysis of fenofibric acid that is a metabolite of fenofibrate [63]. The HPLC system (Waters 2690 alliance, Waters, Milford, MA, USA) was equipped with an autosampler and photodiode array UV detector (Waters^TM^ 996, Waters, Milford, MA, USA). The previously reported HPLC analysis method was used to analyze serum drug concentration [62]. The C-18 reverse phase column was used as the stationary phase (Xterra^TM^ RP-18 5 μm, Waters, Milford, MA, USA). The mobile phase (pH 3.4) was composed of a mixture of 0.02 M phosphate buffer solution and acetonitrile (55:45, *v*/*v*). The flow rate of the mobile phase and UV detection wavelength were 1.2 mL/min and 286 nm, respectively. Calibration samples were prepared as described above, with the exception that, instead of 200 μL of a serum sample, a mixture of stock solution (20 μL) and blank rat serum (180 μL) were used. Calibration curves were constructed in the range of 0.5–200 μg/mL for fenofibric acid using the ratio of fenofibric acid and IS. The PK parameters, including maximum plasma concentration (C_max_), time point of maximum plasma concentration (T_max_), and area under the curve from 0 h to 12 h (AUC_0–12_), were calculated from drug plasma concentration-time curve.

#### 2.4.2. Pharmacodynamic (PD) Studies in Sprague–Dawley Rats

The in vivo hypolipidemic efficacy of the SA-SD-processed fenofibrate microparticles was evaluated in comparison to SD-processed fenofibrate microparticles and unprocessed fenofibrate in 6–7 weeks old male Sprague–Dawley rats (Samtaco, Korea). The animals were divided into nine groups of four animals each prior to distribution for the experiment. Hyperlipidemia was induced by intraperitoneal injection of normal saline containing Triton WR 1339 (equivalent to 250 mg/kg body weight) [64]. Intraperitoneal injection of Triton inhibits peripheral lipoprotein lipase enzymes that are responsible for the removal of lipid particles from the body [65]. The injection of Triton resulted in a temporary increase in serum lipid levels. The control group was intraperitoneally injected with normal saline instead of Triton. After induction of hyperlipidemia, 1 mL of suspensions containing fenofibrate powder samples (equivalent to 10 mg/kg body weight as fenofibrate) in water with 0.2% *w/v* methylcellulose was administered to each group via oral administration using oral zonde. Distilled water (1 mL) was administered to the control group instead of the drug sample. Blood samples were collected by retroorbital puncture under light ether anesthesia beginning at 24 h and 48 h. The collected serum samples were analyzed for the measurement of total cholesterol and triglycerides (TG) using an in vitro diagnostic kit (Stanbio Laboratory, Boerne, TX, USA).

## 3. Results and Discussion

### 3.1. Optimization of Fenofibrate Microparticle Formation Using Box–Behnken Design (BBD)

The experimental runs with the independent variables, including the drug/additive solution concentration, CO_2_ injection rate, and additive content based on the BBD and the pharmaceutical evaluation results of microparticles obtained through each run, are presented in the Supplementary Material. It was demonstrated that the BBD is suitable for the exploration of quadratic response surfaces and the construction of a second-order polynomial model, thus helping to understand the effect of various independent formulation or process variables on the particle formation via the SA-SD process using a small number of experimental runs. As shown in Tables S1–S4 and Figures S2–S7, it is obvious that the particle size and distribution (SPAN value) were significantly affected by drug/additive solution concentration and CO_2_ injection rate, as well as the results of screening design. The content of additive has no significant effect on the particle size and distribution of the SA-SD processed surface modified microparticles. In addition, no interaction between drug/additive solution concentration and CO_2_ injection rate was observed. From these results, it can be suggested that the addition of hydrophilic surfactants could not affect the particle formation process of the SA-SD. In addition, two factor interaction between the drug/additive solution concentration and the content of additive was estimated statistically significant effect. 

Through the analysis of the design of the experimental results described in the Supplementary Material, the optimum SA-SD process conditions (solution concentration: 20 mg/g, CO_2_ injection rate: 25 g/min, contents of additives: 5%) with a small particle size and narrow size distribution (small SPAN value) (Figures S8–S13), good wettability, and fast dissolution rate (Figures S14–S16) were determined and selected for further study. In summary, the dissolution efficiency after 30 min (DE_30 min_) was 46.30% for the unprocessed fenofibrate, while it was 62.50%, 60.95%, and 55.19% for the optimized SA-SD-processed fenofibrate-additive microcomposite particles with Sucroester 15 (S8), TPGS (T8), and Myrj 52 (M8), respectively. A dramatic enhancement of the dissolution rate was observed for the SA-SD-processed fenofibrate microparticles. This means that the particle size reduction has dramatic positive effect on the dissolution rate. In addition, these phenomena may be ascribed to the wettability improvement of the SA-SD-processed composite particles that was achieved by the addition of a hydrophilic surfactant. Among the additives used, Sucroester 15 demonstrated the best performance in regard to the enhancement of the dissolution rate, and this may be due to the relatively good dispersity resulting from the relatively large zeta potential value (−23.85 mV) compared to those of TPGS (−10.71 mV) and Myrj 52 (−12.05 mV) (Tables S5–S7, Supplementary Material). SA-SD-processed fenofibrate microparticles are expected to improve the biopharmaceutical performance of orally administered fenofibrate.

### 3.2. Physicochemical Characterization of Fenofibrate-Additive Microcomposite Particles

**Figure 2:**
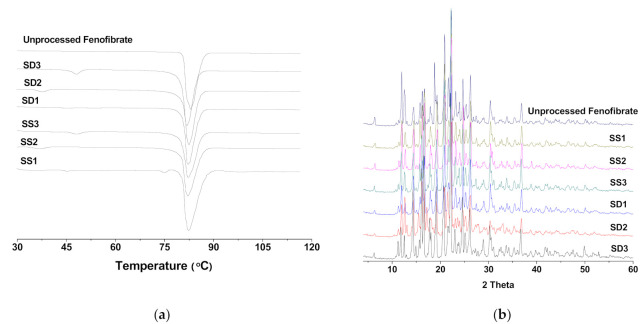
(**a**) DSC thermograms and (**b**) X-ray diffraction patterns of the SA-SD-processed and the SD-processed fenofibrate surface-active additive microcomposite particles.

There was no remarkable difference in the DSC thermogram (showing melting temperature and heat of fusion), PXRD pattern, and FT-IR spectrum among all samples, including raw fenofibrate, and this indicates that raw fenofibrate and all prepared fenofibrate microparticles exist as the same polymorph with similar crystallinity. This result also indicates that the SA-SD processes could not alter the conformational structure of fenofibrate crystals or the SD process (Figure 2).

**Figure 3:**
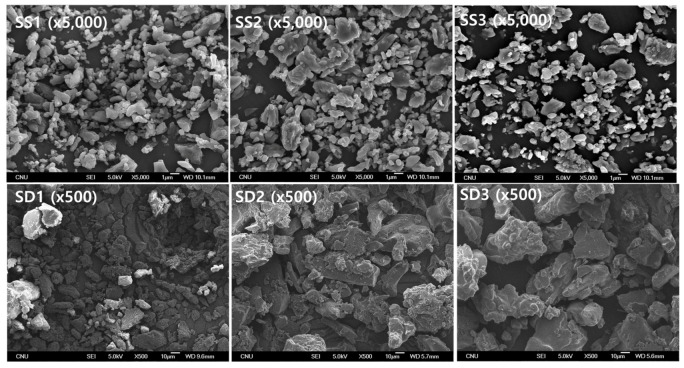
The SEM micrographs obtained by two different particle formation processes, including the SA-SD process (**upper**) and the SD process (**lower**).

**Figure 4:**
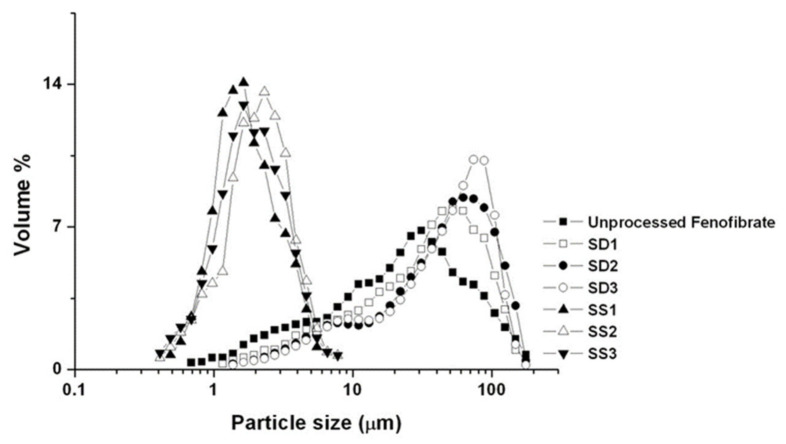
Particle size distribution of the fenofibrate microparticles produced by two different particle formation processes (SD and SA-SD).

The SEM images and particle size distributions are presented in Figure 3 and Figure 4, respectively. As indicated by the SEM images, both the SA-SD and SD processes resulted in irregularly shaped crystals, and no differences were observed in the morphologies. However, the degree of particle size reduction was remarkably different between the SA-SD and SD processes. The SD process produced fenofibrate microparticles possessing mean particle sizes of 33.98 ± 1.21, 43.76 ± 1.53, and 49.16 ± 1.37 μm for SD1, SD2, and SD3, respectively, while the SA-SD process produced particles possessing mean particle sizes of 1.86 ± 0.21, 2.17 ± 0.12, and 2.04 ± 0.25 μm for SS1, SS2, and SS3, respectively. These results indicate that CO_2_ was not only acted as a cosolvent and also acted as an atomizing agent for inducing the formation of smaller and finer particles.

**Figure 5:**
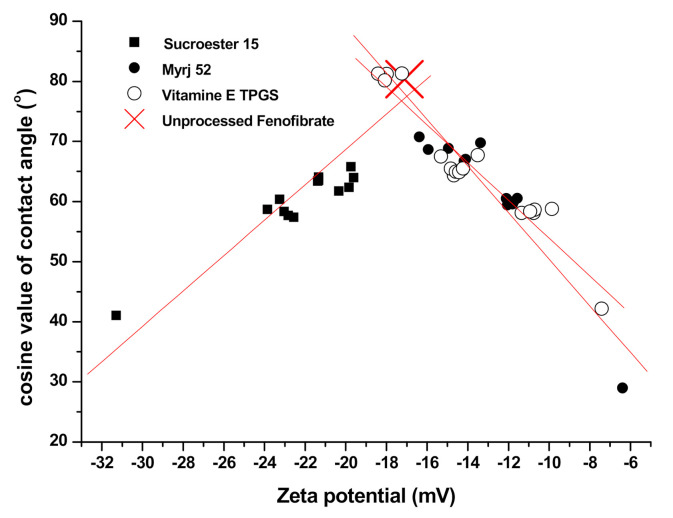
The relationship between zeta potential values and equilibrium contact angles.

The results of the particle size analysis, zeta potential evaluation, and contact angle analysis are summarized in Table 2. While zeta potential values for the SA-SD-processed microparticles were −22.56, −9.87, and −11.74 mV (for SS1, SS2, and SS3, respectively), those of the SD-processed particles were −31.29, −7.42, and −6.39 mV for SD1, SD2, and SD3, respectively. At the same content of each additive, the change in zeta potential (absolute value) was increased in the SD process. The equilibrium contact angles for both the SA-SD- and SD-processed particles were 57.42°, 58.78°, 60.01°, 41.05°, 42.17°, and 28.97° for SS1, SS2, SS3, SD1, SD2, and SD3, respectively. The fenofibrate microparticles produced by the SD process exhibited a lower contact angle than did the composite particles produced by the SA-SD process. To identify the relationship between the zeta potential value and the contact angle, the zeta potential values of all the prepared particles were plotted with the equilibrium contact angle (Figure 5). Plotting the cosine values of the contact angles versus zeta potential values revealed a general trend of decreasing the contact angles with the zeta potential values moving away from −17.12 mV (the zeta potential value of unprocessed fenofibrate), despite a significant scatter around this trend. Therefore, the addition of the surfactant increases the wettability of fenofibrate microparticles. The combination of zeta potential measurement and contact angle measurement, thus, provides useful information on the wettability of the drug/additive composite particles.

**Figure 6:**
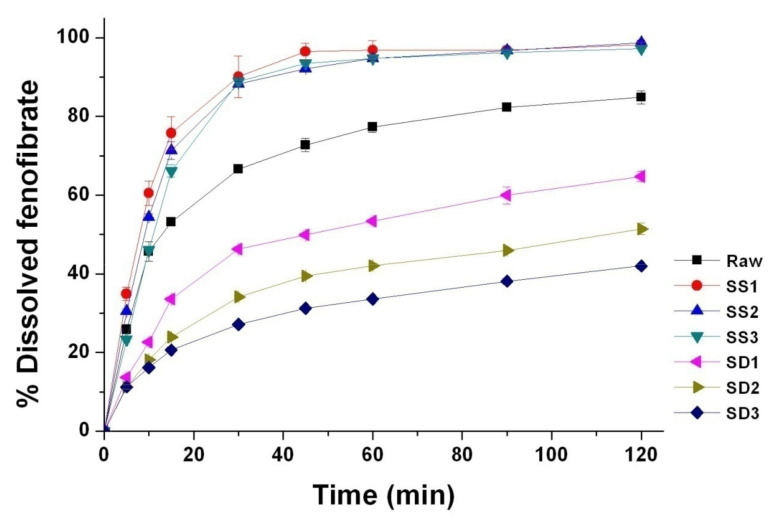
Dissolution profiles of the fenofibrate microcomposite particles with surface-active additives prepared using the SA-SD process and the SD process.

The dissolution profiles of all processed particles are provided in Figure 6. The dissolution efficiencies (DE_30 min_) for the composite particles prepared by the SA-SD process were 63.69%, 60.00%, and 55.84% for SS1, SS2, and SS3, respectively, while the DE_30 min_ values for the composite particles prepared by the SD process were 28.83%, 21.43%, and 18.24% for SD1, SD2, and SD3, respectively. Although the contact angles of the SD-processed particles (SD1, SD2, and SD3) were smaller than were those of the SA-SD-processed particles, the SA-SD-processed particles (SS1, SS2, and SS3) exhibited a more rapid dissolution rate than did the SD-processed particles. The first reason for this result is likely due to the difference in the mean particle sizes between the fenofibrate microparticles prepared by the two different particle formation processes based on the Noyes–Whitney equation [66,67,68]. Thus, it can be also suggested that there is a limit to enhancing the dissolution rate of poorly water-soluble drugs by improving the wettability without particle size reduction.

**Figure 7:**
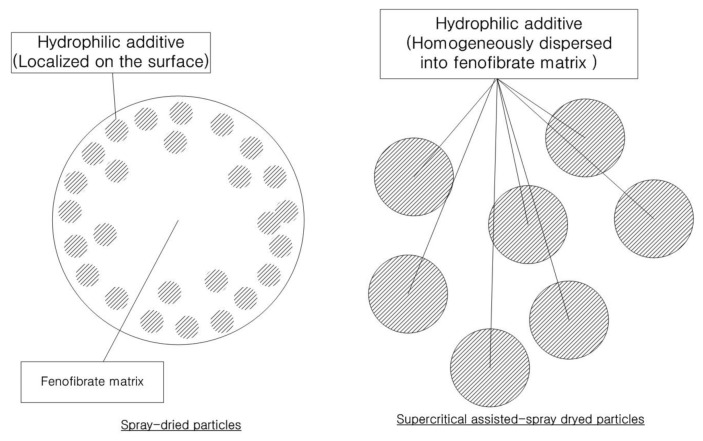
The schematic representation of the fenofibrate microparticles prepared using the SA-SD process and the SD process.

The second presumed cause is the difference in the distribution of surface-active additives between the surface and the inside of the particle matrix. From the results of the zeta potential analysis, it was assumed that the significant differences in zeta potential between the SA-SD-processed and the SD-processed microparticles with the same theoretical formulation may be due to the difference in the actual composition of the surface-active additive distributed on the particle surface that directly contacts the water (Figure 7). It is likely that the surface-active additive composition on the surface layer of the SD-processed particles with better wettability would be higher compared to that of the SA-SD-processed composite particles. Additionally, it should be noted that the hydrophilic additive is present theoretically as a minor component in terms of the weight fraction present in the formed fenofibrate/additive microcomposite particles. The result of possessing an excessive additive amount on the surface may indicate that a relatively much smaller amount of additive is distributed within the SD-processed microparticle. Considering that the initial dissolution step can be dominated by the dissolution rate of the hydrophilic additives rather than by poorly water-soluble drugs, the surface-active excipients that are excessively distributed on the surface will become depleted more rapidly by dissolving into the bulk phase medium during the wetting process at the very beginning of dissolution. Immediately afterward, the creation of a surface layer rich in poorly water-soluble fenofibrate will result in a decrease in the dissolution rate. In contrast, the hydrophilic additive molecules can be dispersed homogeneously in the case of the SA-SD-processed composite particles. As shown in Figure 7, when solution droplets are formed during the SD process, the surface active material are arranged at the interface between the droplets and air and, thus, tend to form surface active material-enriched surface of the dried particles [69,70]. In contrast, it is estimated that the use of ethanol as a co-solvent with the SC-CO_2_ in the SA-SD technology can induce a more homogeneous distribution of surface active material and drug throughout the droplet. In addition, the formation of smaller droplets by the secondary atomization action of SC-CO_2_ dissolved inside the droplet may have influenced these results. Thus, the enhanced wettability could be maintained by the constant amount of hydrophilic additive exposed to the dissolution medium throughout the overall dissolution procedure. The carrier-controlled dissolution model suggested by Corrigan [71] described that the dissolution rate of the minor component is determined by that of another component in excess. This theory supports the above hypothesis by considering the dissolution of a binary component system consisting of fenofibrate and surface-active material [72,73]. 

Furthermore, the XPS results confirm that the above assumption is reasonable. XPS was performed to quantify the amount of hydrophilic additives on the particle surfaces of six different binary composite particles. The theoretical and experimentally measured surface compositions of the prepared composite particles are listed in Table 3. The relative surface compositions and surface coverage values for each of the six formulas are listed in Table 4. Each component within the microparticle samples can be analyzed according to the specific ratio between the different elements. Analysis of the relative amounts of the different elements in the pure materials and in the composite particles can help to estimate the surface composition of particles, particularly for the powder samples that are covered with an additive thin layer. The quantification of surface composition can be achieved using the following method. 

If a particle contains *i* components, at least *one* element is required in the sample to estimate the relative component coverage. *n* is the denotation of elements, and the relative amount of element *n* in pure component *i* and sample are denoted as $I_{\mathrm{component}\;i}^{n}$ and $I_{\mathrm{sample}}^{n}$, respectively. The${\;\gamma }_{i}$ is the relative coverage of component *i*. The following matrix formula can be used to determine the relative coverage of the different components.

(1) $$\left(\begin{array}{ccc} I_{\mathrm{component}\;1}^{1} & \ldots & I_{\mathrm{component}\;i}^{1}\\ \ldots & & \ldots \\ I_{\mathrm{component}\;1}^{n} & \ldots & I_{\mathrm{component}\;i}^{n} \end{array}\right)\left(\begin{array}{c} \gamma_{1}\\ \ldots \\ \gamma_{i} \end{array}\right)=\left(\begin{array}{c} I_{\mathrm{sample}}^{1}\\ \ldots \\ I_{\mathrm{sample}}^{n} \end{array}\right)$$

or

(2) $${Ι}_{\mathrm{component}}\times \Gamma =I_{\mathrm{sample}}$$

The equation is solved by:

(3) $${Ι}_{\mathrm{component}}\times I_{\mathrm{component}}^{-1}=\Gamma$$

In this study, fenofibrate microcomposite particles are composed of fenofibrate and a hydrophilic additive. Fenofibrate contains 20 atoms of carbon (C), four atoms of oxygen (O), and one atom of chloride (Cl). Sucroester 15 (M.W. 580.71) contained 28 carbon atoms and 12 oxygen atoms. TPGS (M.W. 1513) contained 57 C atoms and 28 O atoms. Myrj 52 contains 122 C atoms and 54 O atoms. The relative amount of element n from the components was designated $I_{\mathrm{fenofibrate}}^{n}$ for fenofibrate and $I_{\mathrm{additive}}^{n}$ for each additive.

For each of the common elements C and O in the composite particles, the relative amount can be expressed as

(4) $$I_{\mathrm{sample}}^{C}={\;I}_{\mathrm{fenofibrate}}^{C}\times \gamma_{\mathrm{fenofibrate}}+I_{\mathrm{additive}}^{C}\times \gamma_{\mathrm{additive}}$$

(5) $$I_{\mathrm{sample}}^{O}={\;I}_{\mathrm{fenofibrate}}^{O}\times \gamma_{\mathrm{fenofibrate}}+I_{\mathrm{additive}}^{O}\times \gamma_{\mathrm{additive}}$$

where $I_{\mathrm{sample}}^{C}$ and $I_{\mathrm{sample}}^{O}$ are the relative amounts of C and O in the fenofibrate-additive microparticles, and $\gamma_{\mathrm{fenofibrate}}$ and $\gamma_{\mathrm{additive}}$ are the fractions of the area covered with fenofibrate and the additive, respectively. By reducing two linear equations with two variables, $\gamma_{\mathrm{fenofibrate}}$ and $\gamma_{\mathrm{additive}}$ were calculated [74]. 

**Figure 8:**
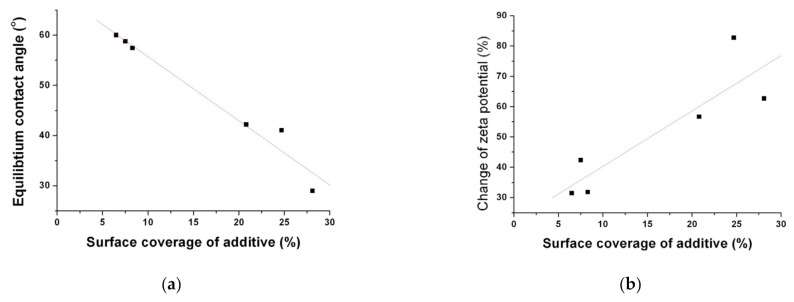
The relationships (**a**) between the surface coverage (%) of the additive and the equilibrium contact angle and (**b**) between the surface coverage (%) of the additive and the % change of the zeta potential.

The calculated surface coverage values of each formula were 8.3%, 7.5%, 6.5%, 24.7%, 20.8%, and 28.1% for SS1, SS2, SS3, SD1, SD2, and SD3, respectively. As expected, it is clear that the surface-active additive incorporated in the fenofibrate microparticles produced by the SD process is more localized on the surface of particles compared to that of SA-SD-processed microparticles. As can be observed in Figure 6 and Table 2, the use of a hydrophilic additive altered the surface characteristics, such as zeta potential and contact angle. To investigate the relationship between the surface coverage of the additive and the wettability of the fenofibrate-additive microcomposite particles, the surface coverage values for all composite particles were plotted with the zeta potential and the contact angles, respectively (Figure 8a,b). Linear correlations were observed (Figure 8). These linear relationships also support the assumption that the hydrophilic additive incorporated into a composite particle produced by the SD process is localized on the particle surface. 

Based on the data described above, it is suggested that the drying kinetics of the SA-SD process and the conventional SD process are different. In the SD process, the driving force for droplet formation is the pneumatic atomization of air. In contrast, fine droplet size was achieved by the combination of atomizing air with CO_2_ in the SA-SD process. Thus, the rapid evaporation and mass transfer of ethanol that was used as solvent from the sprayed droplet occurred in the SA-SD process due to the difference in the spray droplet size and the heated atomization air (Figure 1b). Based on the introduction of CO_2_ for the SA-SD process, fenofibrate microparticles possessing mean particle sizes ranging from 1.86–2.17 μm could be obtained from the SA-SD process. Additionally, due to the rapid evaporation of the solvent, fenofibrate microparticles possessing a homogeneous distribution of surface-active additives could be produced by the SA-SD process.

**Figure 9:**
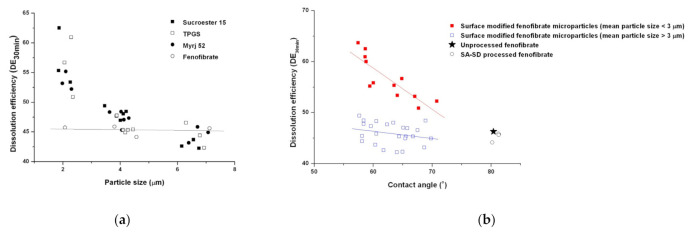
Scatter plots indicating the relationship of the dissolution efficiency versus (**a**) the mean particle size and (**b**) the wettability.

**Figure 10:**
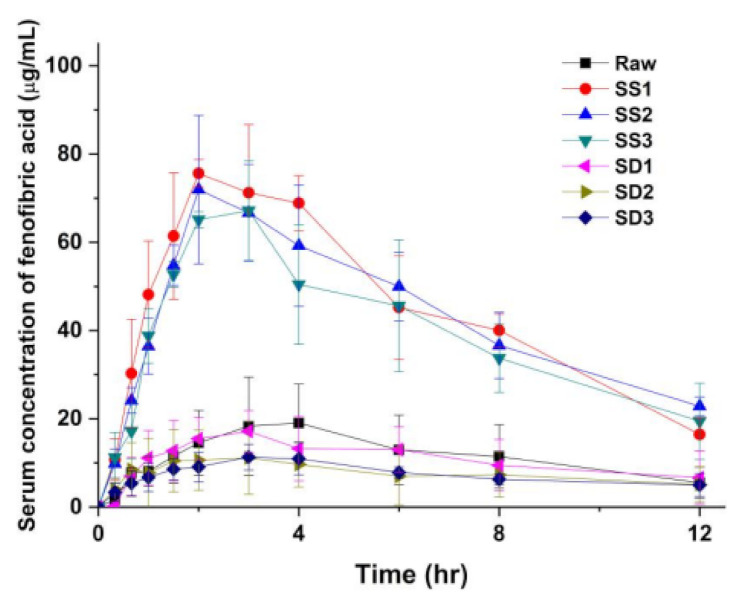
Serum concentration-time profiles of fenofibric acid after single dosing peroral administration of 50 mg/kg in rats. Six different formulations were tested: SS1, SS2, SS3, SD1, SD2, and SD3 (mean ± S.D., *n* = 4).

To evaluate the effect of physical modifications, such as the particle size reduction and the improvement of the wettability on the enhancement of the dissolution rate, DE_30 min_ values for all processed particles were plotted with the mean particle size and contact angle, respectively (Figure 9 and Figure 10). As shown in Figure 9a, in the case of particle size reduction without the addition of a hydrophilic surfactant, the enhancement of the dissolution rate was not observed. However, a dramatic enhancement in the dissolution rate was observed for the SA-SD-processed fenofibrate microparticles with surface-active excipients. The relationship between the dissolution efficiency and contact angle is presented in Figure 9b. The same trend was observed in the correlation study between the contact angle and the dissolution efficiency. Consequently, particle size reduction and improvement of the wettability must be achieved simultaneously to improve the dissolution rate of fenofibrate.

### 3.3. Pharmacokinetic (PK) Profile of Fenofibrate Microparticles in Sprague–Dawley Rats

To investigate the effect of the enhanced dissolution rate of the SA-SD-processed fenofibrate/additive particles on the oral bioavailability of fenofibrate, pharmacokinetic evaluations following an oral dose of 50 mg/kg of unprocessed fenofibrate and of the SA-SD processed (SS1, SS2, and SS3) and the SD-processed particles (SD1, SD2, and SD3) were performed on male Sprague–Dawley rats. Figure 10 presents the pharmacokinetic profiles for each sample, and the pharmacokinetic parameters following noncompartmental analysis are summarized in Table 5. The fenofibrate/additive composite particles from the SA-SD process significantly increased the AUC_0–12 h_ and C_max_ compared to those values after treatment with unprocessed fenofibrate. Unfortunately, the particles from the SD process exhibited no differences compared to the results obtained from the unprocessed fenofibrate. Fenofibrate is a BCS class II drug possessing a high dose number [64,75]. Thus, it is expected that enhanced oral bioavailability would be observed in the case of SA-SD-processed fenofibrate microparticles.

The administration of unprocessed fenofibrate resulted in an AUC_0–12 h_ value of 139.1 ± 74.4 μg∙h/mL and a C_max_ of 19.8 ± 10.1 μg/mL. When the same dose of SA-SD processed fenofibrate microparticles was administrated, the systemic exposure to fenofibrate was increased significantly as reflected in AUC_0–12 h_ values of 537.4 ± 90.1, 519.0 ± 65.5, and 475.1 ± 96.8 μg∙h/mL for SS1, SS2, and SS3, respectively. These pharmacokinetic profiles of fenofibrate in Sprague–Dawley rats reflect the in vitro dissolution rate enhancement achieved by the SA-SD process and the addition of hydrophilic additives. This assumption is also supported by the decrease in T_max_ (3.6 ± 0.6 h for unprocessed fenofibrate, 2.5 ± 0.6, 2.8 ± 1.0, and 2.5 ± 0.6 h for SS1, SS2, and SS3, respectively). The administration of the SD-processed fenofibrate microparticles resulted in AUC_0–12 h_ values of 129.8 ± 59.0, 92.3 ± 56.6, and 89.2 ± 24.9 for SD1, SD2, and SD3, respectively, thus indicating significantly lower bioavailability compared to that of SA-SD-processed microcomposite particles. Additionally, the determined C_max_ values were in the order of SS1 > SS2 > SS3 > unprocessed fenofibrate > SD1 > SD2≒SD3. From the results of the pharmacokinetic evaluation, it was demonstrated that the oral bioavailability of fenofibrate was severely affected by the in vitro dissolution rate of fenofibrate microparticles.

**Figure 11:**
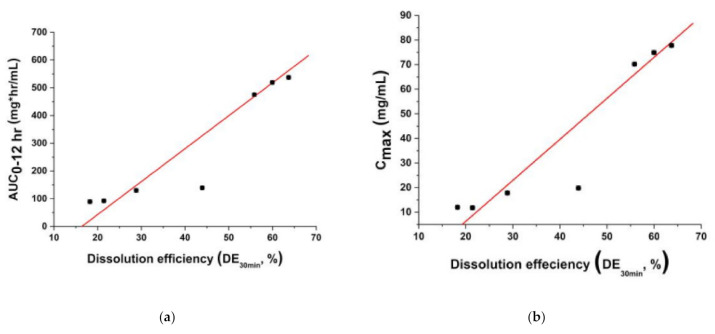
Relationship (**a**) between dissolution efficiency and AUC_0–12 h_ and (**b**) between dissolution efficiency and C_max_. A linear fit is presented as a dashed line that is intended as a guide for the eye and to indicate the relationship presented in the plots.

To investigate the relationship between the in vitro dissolution rate and oral bioavailability of fenofibrate, dissolution efficiency values were plotted with AUC_0–12 h_ values and C_max_ values, respectively (Figure 11a,b). These figures clearly illustrate the correlation between the oral bioavailability of fenofibrate and the in vitro dissolution rate. At dissolution efficiencies (DE_30 min_) ranging from 18.2% to 63.7%, the AUC_0–12 h_, and C_max_ increased with dissolution efficiency, and the relationships were almost linear for both AUC_0–12 h_ and C_max_. These results strongly suggest that the enhanced dissolution rate of fenofibrate microparticles could provide an efficient means to improve the oral bioavailability of fenofibrate.

### 3.4. Pharmacodynamic (PD) Therapeutic Efficacy in Sprague–Dawley Rats

Fenofibrate is a drug that is used to treat hyperlipidemia and is reportedly effective at low doses [76]. The lipid-lowering effect of fenofibrate is known to be dose-dependent. Thus, in the present study the effect of the dissolution rate enhancement on the pharmacodynamic effect of fenofibrate was evaluated in Sprague–Dawley rats. Triton-induced hyperlipidemic rats were used to evaluate the hypolipidemic effect of fenofibrate microparticles. Triton-induced hyperlipidemia can be divided into two stages that include phase I and phase II. In phase I, serum lipid levels were increased and peaked after approximately 24 h of Triton injection. Phase II was the period for recovering normal lipid levels and lasted another 24 h. As fenofibrate possesses a long biological half-life (approximately 20 h), the lipid-lowering effect of the fenofibrate microparticles was monitored for 48 h after the Triton injection. 

The serum lipid profiles in phase I (24 h) and phase II (48 h) for all tested samples are presented in Table 6. Within the phase I period, unprocessed fenofibrate reduced serum triglyceride and cholesterol levels by 54.0% and 70.8%, respectively. In the case of the groups that were administered SA-SD-processed fenofibrate microparticles, serum triglyceride and cholesterol levels were reduced by 81.3–91.3% and 85.9–91.6%, respectively. As expected, no drastic enhancement of the in vivo performance of the SD-processed fenofibrate microparticles was observed. From the serum lipid profiles obtained in phase II, the SA-SD group maintained the lipid-lowering effect (76.6–83.7% inhibition for serum triglyceride and 86.9–93% inhibition for serum cholesterol). These results suggest that the improved dissolution rate of the SA-SD-processed fenofibrate microparticles could enhance the lipid-lowering effect rate in addition to the oral bioavailability of fenofibrate.

## 4. Conclusions

In this study, the SA-SD particle formation technique was applied to functionalize microcomposite particles of fenofibrate (a poorly water-soluble drug) with surface-active additives, and these particles were then compared to those prepared using a conventional SD process. The SA-SD process resulted in a significant decrease in mean particle size compared to the sizes of unprocessed fenofibrate particles and microparticles prepared using the SD process. In particular, more homogeneous composite particles were obtained by the SA-SD process compared to those prepared using the SD process. Additionally, it was also demonstrated that the particle size reduction and the improvement of the wetting property enabled by SA-SD processing with surface-active additives can lead to improved in vitro and in vivo performances in regard to the dissolution rate, the oral bioavailability, and, consequently, the lipid-lowering effect of fenofibrate. In contrast, SD processing did not reduce the size of fenofibrate microcomposite particles, although the wettability of particles was greatly improved. Additionally, the creation of a surface layer that is rich in poorly water-soluble fenofibrate after rapid depletion of excessive surface-active additives distributed locally on the surface of SD-processed particles resulted in a decrease in the dissolution rate. This negative effect on dissolution contributed to the absence of a marked effect on the improvement of PK parameters and PD therapeutic efficacy in vivo. Therefore, it was concluded that the SA-SD process is a superior tool for the micronization of poorly water-soluble drugs to improve their pharmaceutical performance in regard to both PK and PD. 

## Figures and Tables

**Table 1:** The formulations of the fenofibrate microparticles with surface-active additives.

Formula	Surface-Active Additive	Content (*w*/*w*, %) of Additive in Solid Formula	Particle Formation Process
SS1	Sucroester 15	5	Supercritical assisted spray-drying (SA-SD)
SS2	TPGS	5	SA-SD
SS3	Myrj 52	5	SA-SD
SD1	Sucroester 15	5	Spray-drying (SD)
SD2	TPGS	5	SD
SD3	Myrj 52	5	SD

**Table 2:** The results of particle size, zeta potential, and contact angle analyses (mean ± S.D., *n* = 3).

Formula	Mean Particle Size (μm)		SPAN ^a^	Zeta Potential (mV)		Contact Angle (°)	
Raw ^b^	23.80	±0.64	4.25	−17.12	±1.13	80.38	±2.83
SS1	1.86	±0.21	1.27	−22.56	±1.74	57.42	±4.12
SS2	2.17	±0.12	1.18	−9.87	±1.35	58.78	±3.37
SS3	2.04	±0.25	1.22	−11.74	±2.03	60.01	±2.81
SD1	33.98	±1.21	3.84	−31.29	±1.96	41.05	±3.24
SD2	43.76	±1.53	3.93	−7.42	±2.14	42.17	±4.56
SD3	49.16	±1.37	3.60	−6.39	±1.94	28.97	±3.89

^a^ SPAN value was calculated as $\left|\frac{d_{90\%}-d_{10\%}}{d_{50\%}}\right|$. ^b^ Unprocessed fenofibrate.

**Table 3:** Atomic concentrations (%) determined by the XPS and theoretical values for the additives used for preparing the fenofibrate microparticles (mean ± S.D., *n* = 3).

	Numbers of Atom			Atomic Concentration (%)					
Substance	C	O	Cl	C*1s*		O*1s*		Cl*2p*	
Fenofibrate	20	4	1	80.4	±0.8	15.0	±0.7	4.6	±0.4
Theory ^a^	-	-	-	80.0		16.0		4.0	
Sucroester 15	28	12	-	70.3	±0.7	29.7	±0.5	-	
Theory ^a^	-	-	-	70.0		30.0		-	
TPGS	57	28	-	68.1	±0.9	31.9	±0.6	-	
Theory ^a^	-	-	-	67.1		32.9		-	
Myrj 52	122	54	-	69.9	±0.7	20.1	±0.4	-	
Theory ^a^	-	-	-	69.3		30.7		-	

^a^ The theoretical values for all substances were calculated using the molecular weight and empirical molecular formula.

**Table 4:** Atomic concentrations (%) determined by XPS for six different composite particles (mean ± S.D., *n* = 3).

Formula	Atomic Concentration (%)						Surface Coverage of Additive (%)	Surface Excess of Additive ^a^
	C1s		O1s		Cl2p			
SS1	77.5	±0.9	16.8	±0.6	3.7	±0.6	8.3	1.7
SS2	78.7	±0.6	17.2	±0.4	3.6	±0.3	7.5	1.5
SS3	78.1	±0.5	16.7	±0.5	4.1	±0.7	6.5	1.3
SD1	77.7	±0.4	19.5	±0.7	2.8	±0.8	24.7	4.9
SD2	77.9	±0.8	19.7	±0.3	2.4	±0.9	20.8	4.2
SD3	77.2	±0.7	20.2	±0.6	2.6	±0.7	28.1	5.6

^a^ Calculated as: (surface coverage of additive)/(the theoretical %weight fraction of additive, 5%).

**Table 5:** Pharmacokinetic parameters of unprocessed fenofibrate and the SA-SD-processed and the SD-processed fenofibrate-additive microparticles after oral administration in Sprague–Dawley rats (mean ± S.D.; *n* = 4).

	AUC_0–12 h_ (μg∙h/mL)		C_max_ (μg/mL)		T_max_ (h)	
Unprocessed Fenofibrate	139.1	±74.4	19.8	±10.1	3.5	±0.6
SS1	537.4	±90.1	77.8	±6.2	2.5	±0.6
SS2	519.0	±65.5	74.9	±14.3	2.8	±1.0
SS3	475.1	±96.8	70.2	±8.9	2.5	±0.6
SD1	129.8	±59.0	17.8	±5.3	3.5	±1.0
SD2	92.3	±56.6	11.8	±6.7	3.3	±0.6
SD3	89.2	±24.9	12.0	±3.6	3.5	±0.6

**Table 6:** The serum lipid profiles in phase I (24 h) and phase II (48 h) for all tested samples (mean ± S.D., *n* = 4) ^a^.

	Phase I (After 24 h)				Phase II (After 48 h)			
Group	Total Cholesterol (mg/dL)		Triglycerides (mg/dL)		Total Cholesterol (mg/dL)		Triglycerides (mg/dL)	
Control	70.4	±9.5	131.7	±6.7	68.9	±10.6	109.2	±4.9
Triton	256.7	±9.0	370.1	±7.5	141.1	±6.2	221.1	±4.5
Unprocessed	124.7	±24.5 (70.8)	241.4	±3.7 (54.0)	88.7	±9.2 (72.6)	159.8	±7.1 (54.8)
SS1	86.1	±8.6 (91.6)	152.4	±2.5 (91.3)	73.9	±4.0 (93.0)	127.5	±3.9 (83.7)
SS2	89.6	±10.8 (89.7)	173.9	±10.5 (82.3)	74.1	±5.9 (92.8)	139.6	±12.2 (72.9)
SS3	96.7	±4.7 (85.9)	170.8	±4.0 (83.6)	78.3	±9.2 (86.9)	135.4	±3.3 (76.6)
SD1	138.1	±1.3 (63.6)	231.9	±5.3 (58.0)	93.6	±9.8 (65.8)	148.7	±5.8 (64.7)
SD2	146.1	±2.3 (59.3)	243.9	±4.1 (52.9)	91.7	±14.0 (68.4)	165.8	±7.6 (49.5)
SD3	146.3	±23.8 (59.2)	249.7	±6.4 (50.5)	102.9	±8.5 (52.9)	159.8	±5.1 (54.8)

^a^ Data in parentheses represent % inhibition calculate as follows; (1-((serum lipid level of each sample group)-(serum lipid level of control group))/((serum lipid level of Triton group)-(serum lipid level of control)))×100.

## Supplementary Materials

The following are available online at https://www.mdpi.com/article/10.3390/pharmaceutics13122061/s1, Figure S1: Chemical Structures of (a) fenofibrate (MW 360.84), (b) d-α-tocopheryl polyethelyene glycol 1000 succinate (TPGS, MW 1513, hydrophilic-lipophilic balance (HLB) 13.2), (c) polyoxyethylene 40 stearate (Myrj 52, MW 2046.58, HLB 16.9) and (d) sucrose monopalmitate (sucroester 15, MW 580.71, HLB 15), Figure S2: Significant standardized main effects on the mean particle size, SPAN, and DE_30 min_ estimated from BBD using Sucroester 15, Figure S3: Significant standardized main effects on the mean particle size, SPAN, and DE_30 min_ estimated from BBD using TPGS, Figure S4: Significant standardized main effects on the mean particle size, SPAN, and DE_30 min_ estimated from BBD using Myrj 52, Figure S5: Response surface plots constructed by the BBD using Sucroester 15 as an additive, Figure S6: Response surface plots constructed by the BBD using TPGS 15 as an additive, Figure S7: Response surface plots constructed by the BBD using Myrj 52 as an additive, Figure S8: The SEM micrographs of the SA-SD processed fenofibrate/Sucroester 15 microcomposite particles, Figure S9: The SEM micrographs of the SA-SD processed fenofibrate/TPGS 15 microcomposite particles, Figure S10: The SEM micrographs of the SA-SD processed fenofibrate/Myrj 52 microcomposite particles, Figure S11: The cumulative particle size distributions of the SA-SD processed fenofibrate/Sucroester 15 microcomposite particles, Figure S12: The cumulative particle size distributions of the SA-SD processed fenofibrate/TPGS 15 microcomposite particles, Figure S13: The cumulative particle size distributions of the SA-SD processed fenofibrate/Myrj 52 microcomposite particles, Figure S14: Dissolution profiles of the SA-SD processed fenofibrate-additive microcomposite particles (fenofibrate/Sucroester 15), Figure S15: Dissolution profiles of profiles of the SA-SD processed fenofibrate-additive microcomposite particles (fenofibrate/TPGS), Figure S16: Dissolution profiles of profiles of the SA-SD processed fenofibrate-additive microcomposite particles (fenofibrate/Myrj 52), Table S1: Experimental runs and observed responses in preparation of fenofibrate/Sucroester 15 microparticles, Table S2: Experimental runs and observed responses in preparation of fenofibrate/TPGS microparticles, Table S3: Experimental runs and observed responses in preparation of fenofibrate/Myrj 52 microparticles, Table S4: Regression equations of the best fitted models, Table S5: Zeta potential values and equilibrium contact angle of the SA-SD processed fenofibrate/Sucroester 15 composite particles, Table S6: Zeta potential values and equilibrium contact angle of the SA-SD processed fenofibrate/TPGS composite particles, Table S7: Zeta potential values and equilibrium contact angle of the SA-SD processed fenofibrate/Myrj 52 composite particles.
