# Supplementary material for: Preparation and Characterization of Fenofibrate Microparticles with Surface-Active Additives: Application of a Supercritical Fluid-Assisted Spray-Drying Process
Source: Pharmaceutics. 2021 Dec 2;13(12):2061. doi: 10.3390/pharmaceutics13122061 (PMC8709266; doi:10.3390/pharmaceutics13122061)
Supplement: Supplementary file 1 [file pharmaceutics-13-02061-s001.zip › pharmaceutics-1461445-supplementary.pdf]

pharmaceutics

MDPI

Supplementary Materials: Preparation and Characterization of Fenofibrate Microparticles with Surface-Active Additives: Application of a Supercritical Fluid-Assisted Spray-Drying Process

Jeong-Soo Kim, Heejun Park, Kyu-Tae Kang, Eun-Sol Ha, In-hwan Baek , Min-Soo Kim and Sung-Joo Hwang

1. Chemical Structures of fenofibrate and used additives

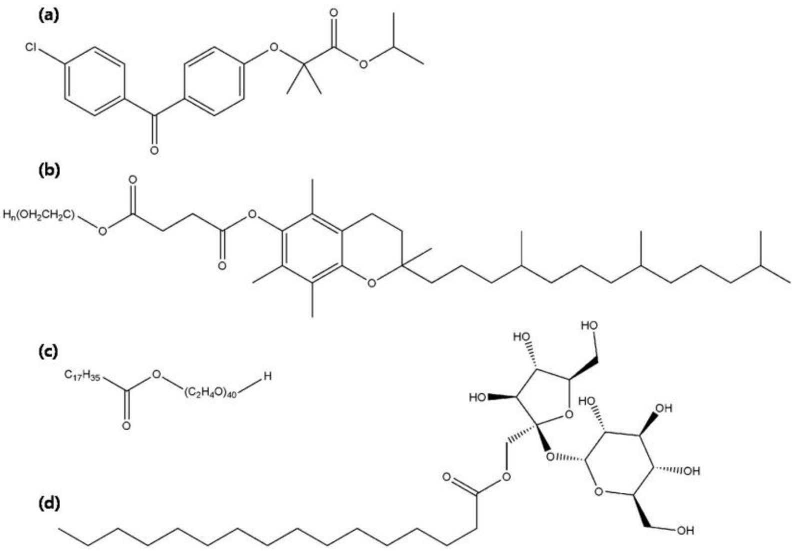

Figure S1. Chemical Structures of (a) fenofibrate (MW 360.84), (b) d-α-tocopheryl polyethelyene glycol 1000 succinate (TPGS, MW 1513, hydrophilic-lipophilic balance (HLB) 13.2), (c) polyoxyethylene 40 stearate (Myrj 52, MW 2046.58, HLB 16.9) and (d) sucrose monopalmitate (sucroester 15, MW 580.71, HLB 15).

2. Experimental runs and observed responses via Box-Behnken design (BBD)

The experimental runs with the independent variables, including the drug/additive solution concentration, the CO2 injection rate and the content of additive, are presented in Table S1, S2 and S3 for Sucroester 15, TPGS and Myrj 52, respectively. In this study, a three-factor, three-level BBD was used and the best fitted models were selected for each response based on the multiple correlation coefficient, adjust multiple correlation coefficient (adjusted R^2) and the predicted residual sum of square (PRESS), provided by Design-Expert software, (Table S4). The lack of fit analysis shows that the selected model is appropriate for the description of all responses. Figure S2, S3 and S4 shows standardized main effects on the observed responses for each additive, respectively, and positive or negative signs mean a positive effect or a negative effect for the factors. Standardized main effect was calculated by dividing the main effects with the standard error of the main effects.

Table S1. Experimental runs and observed responses in preparation of fenofibrate/Sucroester 15 microparticles.

Run	Fenofibrate/Sucroester 15 solution concentration (mg/g)	CO_2 injection rate (g/min)	Contents of additives (%)	Mean particle size (μm)	SPAN [a]	DE_{30min}[b] (%)
Unprocessed fenofibrate	-	-	-	23.80	4.25	46.30
S1	20	15	2.5	2.26	1.39	53.39
S2	50	25	2.5	4.11	1.59	48.05
S3	50	25	2.5	4.01	1.47	46.96
S4	50	15	5	4.21	1.67	48.45
S5	50	35	0	3.80	1.36	45.90
S6	80	25	5	6.56	2.06	43.70
S7	20	25	0	2.07	1.25	45.74
S8	20	25	5	1.87	1.25	62.50
S9	80	35	2.5	6.15	1.93	42.61
S10	80	25	0	7.12	1.85	45.57
S11	50	15	0	4.57	1.60	44.15
S12	50	35	5	3.46	1.42	49.41
S13	80	15	2.5	6.75	2.26	42.25
S14	20	35	2.5	1.85	1.23	55.34
S15	50	25	2.5	3.87	1.59	47.68

[a] SPAN value was calculated as $\left|\frac{d_{90\%}-d_{10\%}}{d_{50\%}}\right|$, [b] DE_{30min} value was calculated as $\frac{\int_0^t y dt}{y_{100} t} \times 100$.

Table S2. Experimental runs and observed responses in preparation of fenofibrate/TPGS microparticles.

Run	Fenofibrate/ TPGS solution concentration (mg/g)	CO_2 injection rate (g/min)	Contents of additives (%)	Mean particle size (μm)	SPAN [a]	DE_{30min}[b] (%)
Unprocessed fenofibrate	-	-	-	23.80	4.25	46.30
T1	20	15	2.5	2.35	1.27	50.90
T2	50	25	2.5	4.27	1.58	45.32
T3	50	25	2.5	4.18	1.52	44.91
T4	50	15	5	4.45	1.69	45.41
T5	50	35	0	3.80	1.36	45.90
T6	80	25	5	6.78	2.06	44.41
T7	20	25	0	2.07	1.25	45.74
T8	20	25	5	2.29	1.30	60.95
T9	80	35	2.5	6.30	1.96	46.55
T10	80	25	0	7.12	1.85	45.62
T11	50	15	0	4.57	1.60	44.15
T12	50	35	5	3.88	1.40	47.77
T13	80	15	2.5	6.93	2.07	42.33
T14	20	35	2.5	2.06	1.21	56.68
T15	50	25	2.5	4.10	1.57	45.34

[a] SPAN value was calculated as $\left|\frac{d_{90\%}-d_{10\%}}{d_{50\%}}\right|$, [b] DE_{30min} value was calculated as $\frac{\int_0^t y dt}{y_{100} t} \times 100$.

Table S3. Experimental runs and observed responses in preparation of fenofibrate/Myrj 52 microparticles.

Run	Fenofibrate/Myrj 52 solution concentration (mg/g)	CO_2 injection rate (g/min)	Contents of additives (%)	Mean particle size (μm)	SPAN [a]	DE_{30min} [b] (%)
Unprocessed fenofibrate	-	-	-	23.80	4.25	46.30
M1	20	15	2.5	2.30	1.37	52.23
M2	50	25	2.5	4.13	1.56	47.07
M3	50	25	2.5	4.06	1.51	45.33
M4	50	15	5	4.30	1.69	47.34
M5	50	35	0	3.80	1.36	45.90
M6	80	25	5	6.70	1.98	45.87
M7	20	25	0	2.07	1.25	45.74
M8	20	25	5	2.10	1.33	55.19
M9	80	35	2.5	6.39	1.92	43.18
M10	80	25	0	7.12	1.85	45.62
M11	50	15	0	4.57	1.60	44.15
M12	50	35	5	3.63	1.39	48.34
M13	80	15	2.5	7.07	2.15	44.92
M14	20	35	2.5	1.98	1.28	53.18
M15	50	25	2.5	4.03	1.59	48.42

[a] SPAN value was calculated as $\left|\frac{d_{90\%}-d_{10\%}}{d_{50\%}}\right|$, [b] DE_{30min} value was calculated as $\frac{\int_0^t y\,dt}{y_{100}t} \times 100$.

Table S4. Regression equations of the best fitted models[a].

Polynomial equation	Lack of fit: Sum of Squares	Lack of fit: P-value
Additive : Sucroester 15		
Mean Particle Size = 4.18+2.32*(Drug/additive solution concentration)-0.32*(CO_2 injection rate)	0.63	0.1803
SPAN = 1.55+0.37*(Drug/additive solution concentration)-0.12*(CO_2 injection rate)+0.12*(Drug/additive solution concentration)^2	0.00	0.8583
DE_{30min} = 48.11-5.36*(Drug/additive solution concentration)+2.84*(Contents of Sucroester 15)-4.66*(Drug/additive solution concentration)* (Contents of Sucroester 15)	22.22	0.0776
Additive : TPGS		
Mean Particle Size = 4.18+2.29*(Drug/additive solution concentration)-0.28*(CO_2 injection rate)+0.31*(Drug/additive solution concentration)^2	0.087	0.2135
SPAN = 1.58+0.36*(Drug/additive solution concentration)-0.09*(CO_2 injection rate)	0.065	0.1296
DE_{30min} = 45.19-4.42*(Drug/additive solution concentration)+2.14*(Contents of TPGS)-4.11*(Drug/additive solution concentration)*(Contents of TPGS)+3.65*(Drug/additive solution concentration)^2	19.60	0.0529
Additive : Myrj 52		

Mean Particle Size = 4.07+2.35*(Drug/additive solution concentration)-0.31*(CO_2 injection rate)-0.10*(Contents of Myrj 52)+0.39*(Drug/additive solution concentration)^2	0.051	0.1302
SPAN = 1.55+0.33*(Drug/additive solution concentration)-0.11*(CO_2 injection rate)+0.11*(Drug/additive solution concentration)^2	0.010	0.3452
DE_{30min} = 47.50-3.34*(Drug/additive solution concentration)+1.92*(Contents of Myrj 52)	44.60	0.3680

[a] Only the terms with statistical significance (P<0.05)are included.

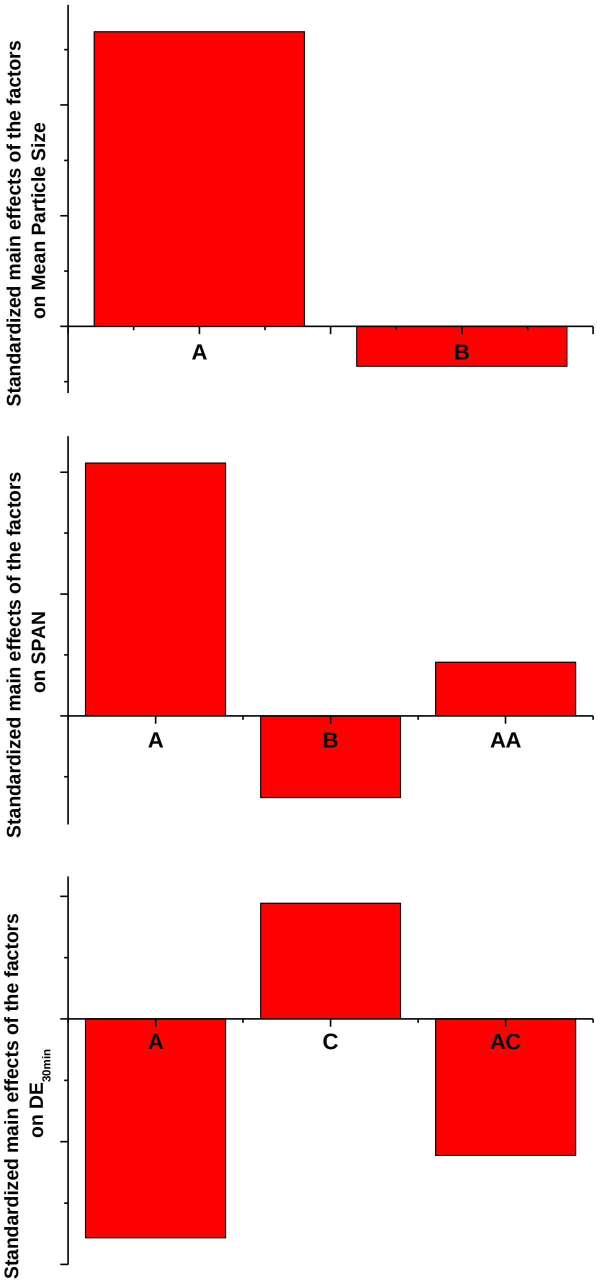

Figure S2. Significant standardized main effects on the mean particle size, SPAN and DE_{30min} estimated from BBD using Sucroester 15.

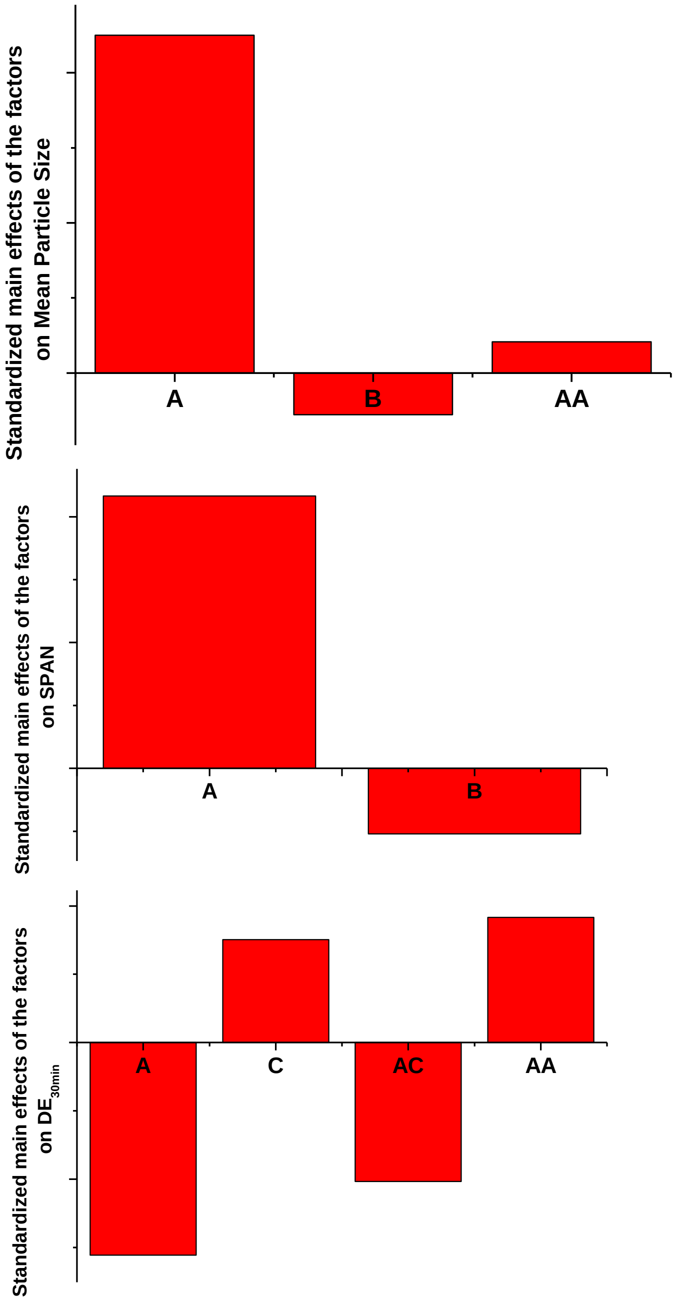

Figure S3. Significant standardized main effects on the mean particle size, SPAN and DE_{30min} estimated from BBD using TPGS.

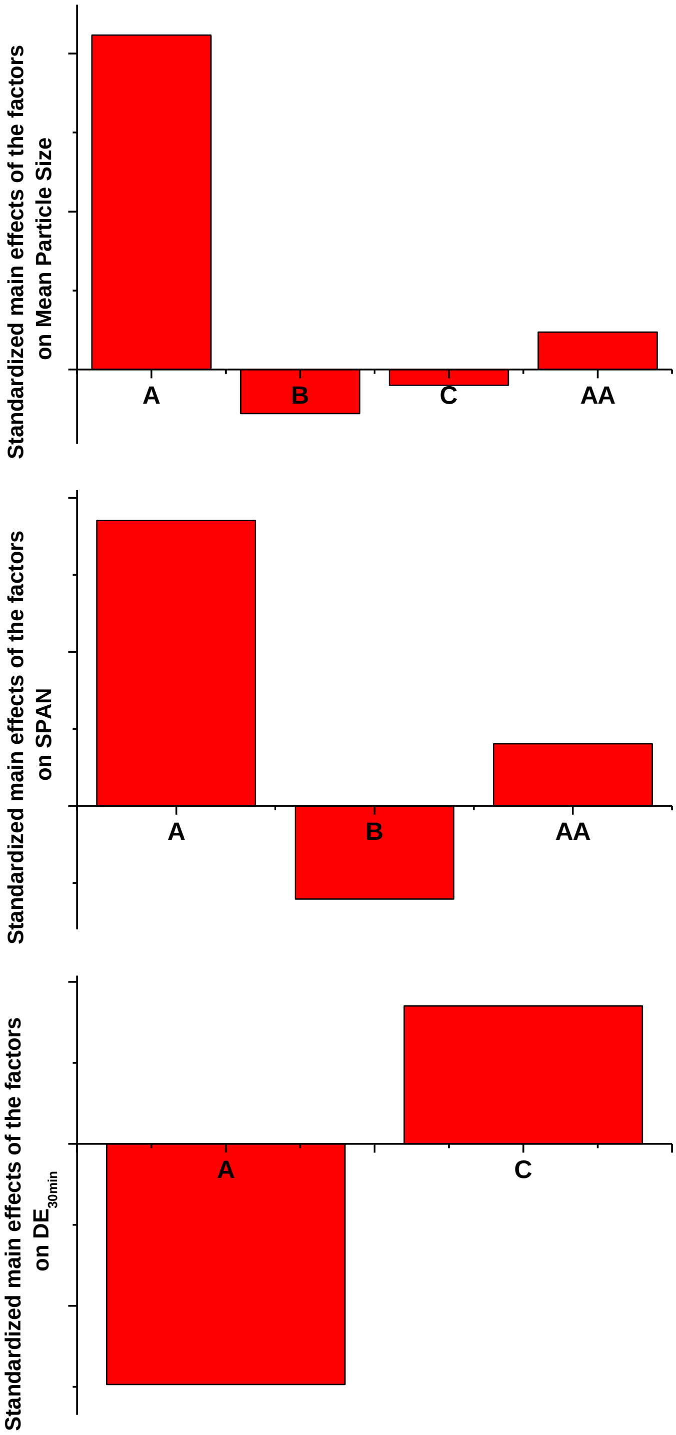

Figure S4. Significant standardized main effects on the mean particle size, SPAN and DE_{30min} estimated from BBD using Myrj 52.

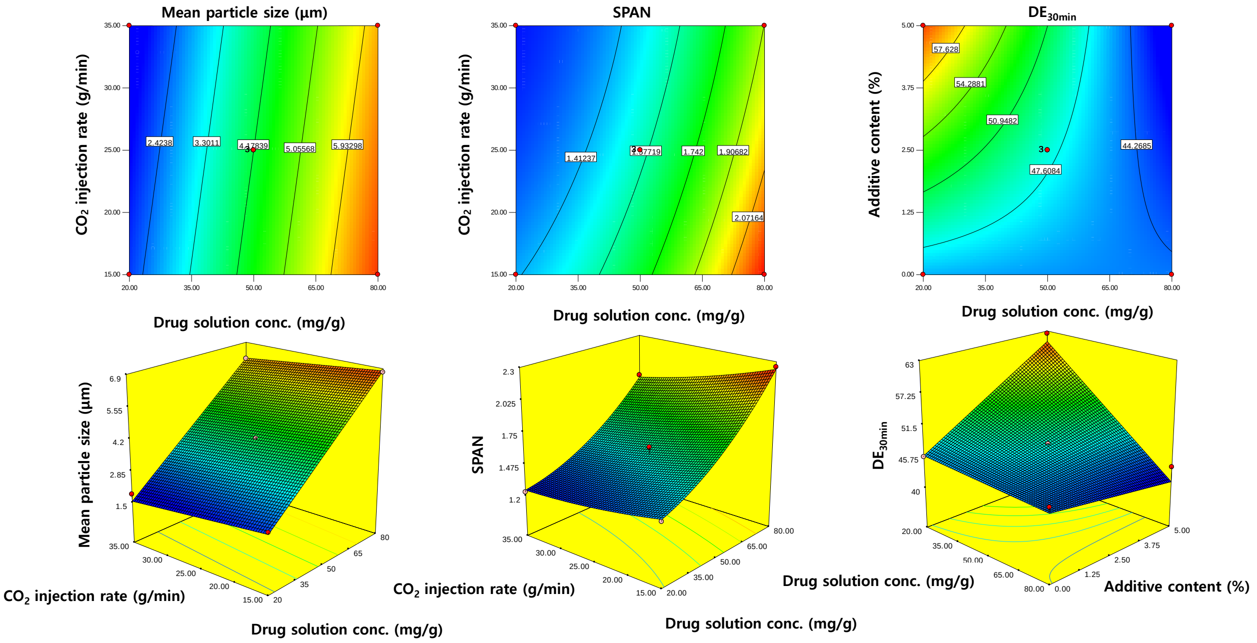

Figure S5. Response surface plots constructed by the BBD using Sucroester 15 as an additive.

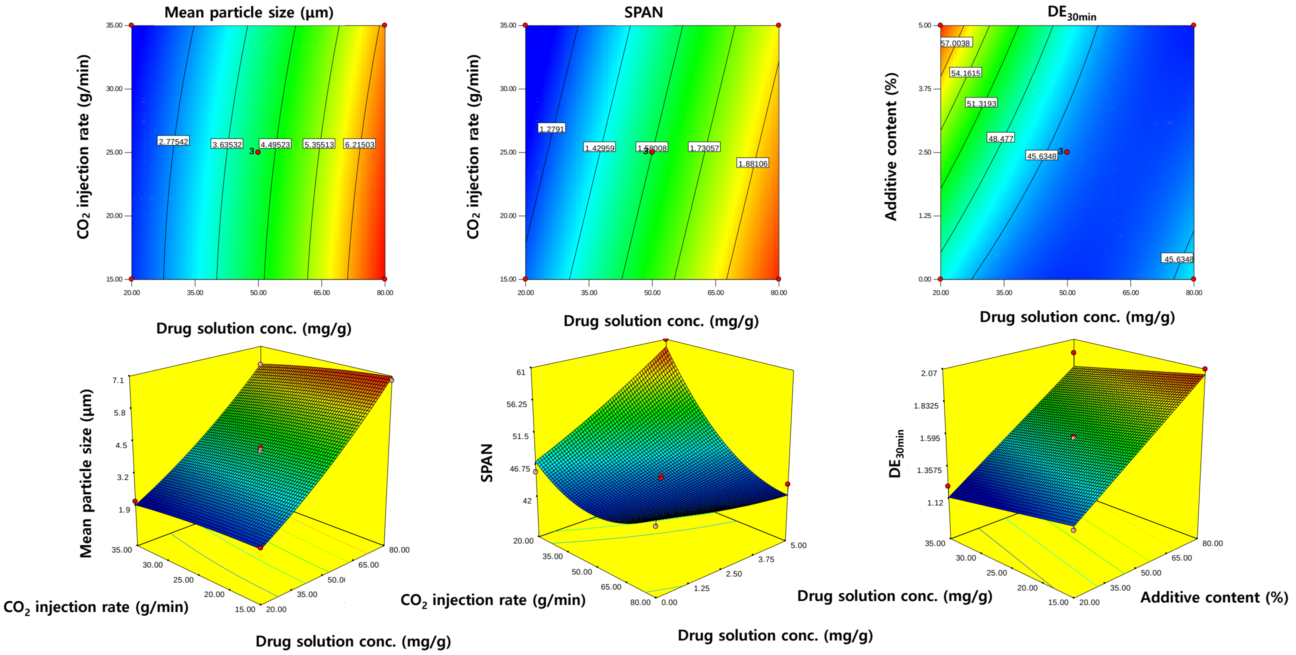

Figure S6. Response surface plots constructed by the BBD using TPGS 15 as an additive.

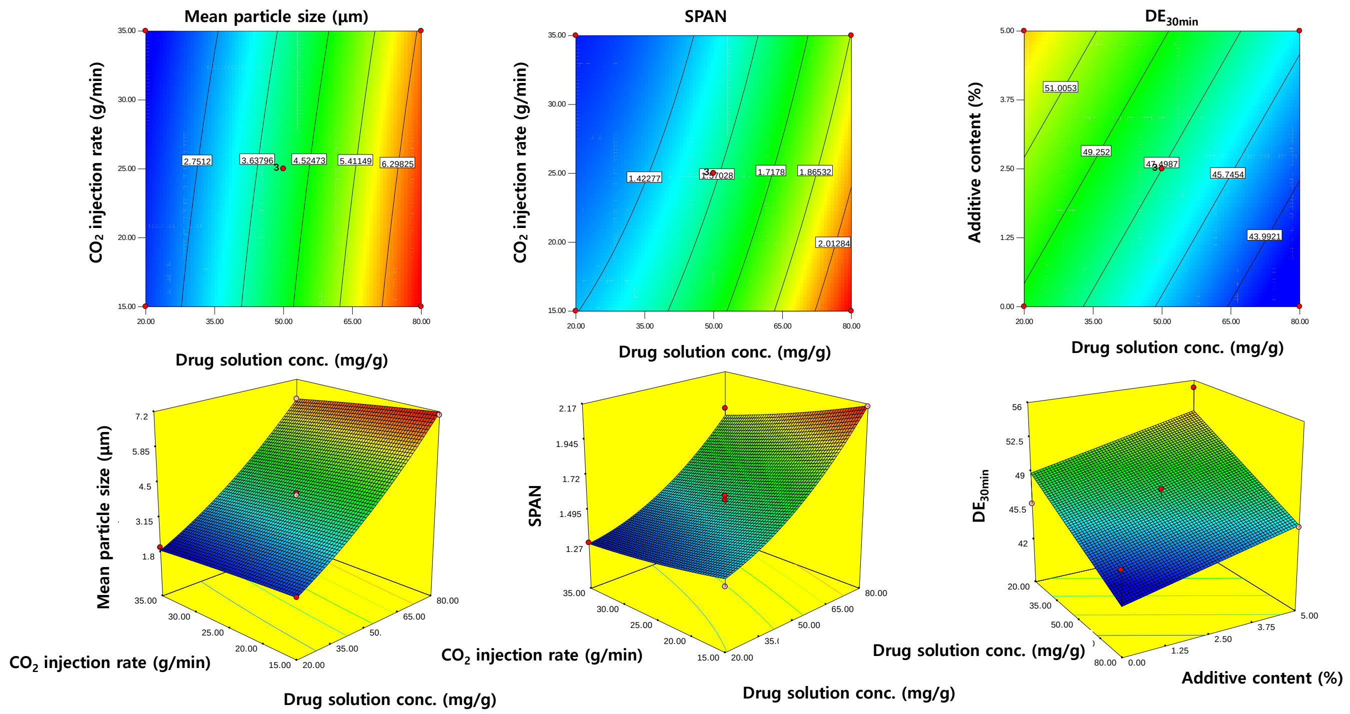

Figure S7. Response surface plots constructed by the BBD using Myrj 52 as an additive.

2. Physicochemical characteristics of fenofibrate-additive microcomposite particles prepared at experimental runs via Box-Behnken design (BBD)

The SEM micrographs and cumulative particle size distributions of SA-SD processed fenofibrate composite particles are presented in Figure S8-S10 and Figure S11-S13, respectively. As indicated form SEM images, no remarkable differences were observed in the particle morphology betweeb micronized fenofibrate particles prepared by the SD and SA-SD processes. In the PXRD patterns, DSC curves and FT-IR spectra, no differences were observed for the SA-SD processed fenofibrate-additive microcomposite particles compared to unprocessed fenofibrate (data not shown). These physicochemical characterization results show that the addition of hydrophilic surfactants could not change the crystalline form of fenofibrate particles.

The results of zeta potential evaluation and contact angle measurement are summarized in Table S5, S6 and S7. The measured zeta potential values of fenofibrate particles without surface active additive prepared by the SA-SD process showed no difference compared to unprocessed fenofibrate. While, the measured zeta potential values of fenofibrate-additive microcomposite particles were slightly increased or decreased compared to unprocessed fenofibrate. In particular, the zeta potential values of fenofibrate/TPGS or fenofibrate/Myrj 52 particles were changed to the neutral region as the contents of additives increased. In contrast, the zeta potential values of the fenofibrate/Sucroester 15 composite particles were more negative as increased the contents of additives. The contact angles of fenofibrate-additive microcomposite particles were decreased as the contents of additives increased. This resut suggests that the addition of hydrophilic surfactant can result in the improvement of the wettability of fenofibrate.

Dissolution profiles of the fenofibrate-additive microcomposite particles are presented in Figure S14, 15 and 16. After 30 min, the dissolved % of the unprocessed fenofibrate was 66%, whereas the dissolved % of fenofibrate from the SA-SD processed particles with surface active additives was 93%, 87% and 88% for S8, T8 and M8, respectively. From results of the dissolution test, it is confirmed that the particle size reduction shows dramatic enhancement on the dissolution rate, if only the improvement of the wettability is ensured. In particular, sucroester 15 showed the best performance in the enhancement

of the dissolution rate. This can be explained by the results of the zeta potential measurements. The zeta potential is a surface character related to the dispersity and the stability of the dispersed system [1-3]. The relatively large zeta potential (in absolute value) is resulted in the strong electro static repulsion between the particles in the dispersed system and, consequently, indicates no tendency for the particles to aggregate. By the addition of TPGS and Myrj 52, the zeta potential (in absolute values) were reduced, whereas the zeta potential (in absolute values) were increased by the addition of Sucroester 15. Consequently, fenofibrate/Sucroester 15 composite particles have relative good dispersity, compared to other composite particles. Therefore, it can be suggested that the combination of the wettability improvement and the relative good dispersity result in the synergistic effect on the dissolution rate enhancement of the SA-SD processed fenofibrate/Sucroester 15 composite particles. The dissolution rate of poorly water-soluble drugs could be a rate-limiting process in drug absorption from a solid dosage form. The SA-SD processed fenofibrate/additive composite partices could enhance the dissolution rate and would improve bioavailability of fenofibrate.

Table S5. Zeta potential values and equilibrium contact angle of the SA-SD processed fenofibrate/Sucroester 15 composite particles (mean ± S.D., n=3).

Sample	Zeta potential (mV)		Contact angle (°)		Sample	Zeta potential (mV)		Contact angle (°)	
Raw[a]	-17.12	± 1.13	80.38	± 2.83	S8	-23.85	± 1.62	58.68	± 3.30
S1	-21.34	± 1.67	64.07	± 2.84	S9	-20.35	± 2.50	61.78	± 2.62
S2	-21.39	± 2.29	63.39	± 3.68	S10	-18.41	± 2.06	81.29	± 4.05
S3	-19.75	± 2.42	65.78	± 3.74	S11	-18.08	± 1.91	80.17	± 4.33
S4	-23.03	± 1.66	58.36	± 2.88	S12	-22.83	± 1.89	57.67	± 4.82
S5	-17.98	± 2.34	81.24	± 2.09	S13	-19.61	± 2.43	63.99	± 3.67
S6	-23.26	± 2.23	60.38	± 2.94	S14	-21.33	± 2.18	63.54	± 4.75
S7	-17.24	± 1.68	81.31	± 4.65	S15	-19.84	± 1.99	62.35	± 4.61

[a] Unprocessed fenofibrate.

Table S6. Zeta potential values and equilibrium contact angle of the SA-SD processed fenofibrate/TPGS composite particles (mean ± S.D., n=3).

Sample	Zeta potential (mV)		Contact angle (°)		Sample	Zeta potential (mV)		Contact angle (°)	
Raw[a]	-17.12	± 1.13	80.38	± 2.83	T8	-10.71	± 2.19	58.63	± 4.14
T1	-13.51	± 1.74	67.69	± 2.42	T9	-15.32	± 1.58	67.50	± 2.34
T2	-14.68	± 1.43	64.36	± 3.23	T10	-18.41	± 2.06	81.29	± 4.05
T3	-14.83	± 1.33	65.45	± 3.46	T11	-18.08	± 1.91	80.17	± 4.33
T4	-10.76	± 2.17	58.12	± 3.21	T12	-10.93	± 2.39	58.37	± 3.14
T5	-17.98	± 2.34	81.24	± 2.09	T13	-14.58	± 2.05	64.98	± 2.30
T6	-11.34	± 1.66	58.11	± 4.22	T14	-14.43	± 2.16	64.87	± 2.12
T7	-17.24	± 1.68	81.31	± 4.65	T15	-14.23	± 1.89	65.52	± 2.53

[a] Unprocessed fenofibrate.

Table S7. Zeta potential valus and equilibrium contact angle of the SA-SD processed fenofibrate/Myrj 52 composite particles (mean ± S.D., n=3).

Sample	Zeta potential (mV)		Contact angle (°)		Sample	Zeta potential (mV)		Contact angle (°)	
Raw[a]	-17.12	± 1.13	80.38	± 2.83	M8	-12.05	± 1.79	59.44	± 4.30
M1	-16.38	± 1.68	70.77	± 2.26	M9	-15.94	± 1.71	68.66	± 4.02
M2	-14.22	± 1.66	65.12	± 2.25	M10	-18.41	± 2.06	81.29	± 4.05
M3	-14.18	± 2.23	66.75	± 4.74	M11	-18.08	± 1.91	80.17	± 4.33
M4	-11.80	± 2.36	59.60	± 2.06	M12	-11.57	± 2.16	60.54	± 3.03
M5	-17.98	± 2.34	81.24	± 2.09	M13	-13.38	± 1.57	69.79	± 2.43
M6	-12.10	± 1.55	60.52	± 3.95	M14	-14.11	± 1.71	67.04	± 3.35
M7	-17.24	± 1.68	81.31	± 4.65	M15	-14.95	± 1.98	68.88	± 3.93

[a] Unprocessed fenofibrate.

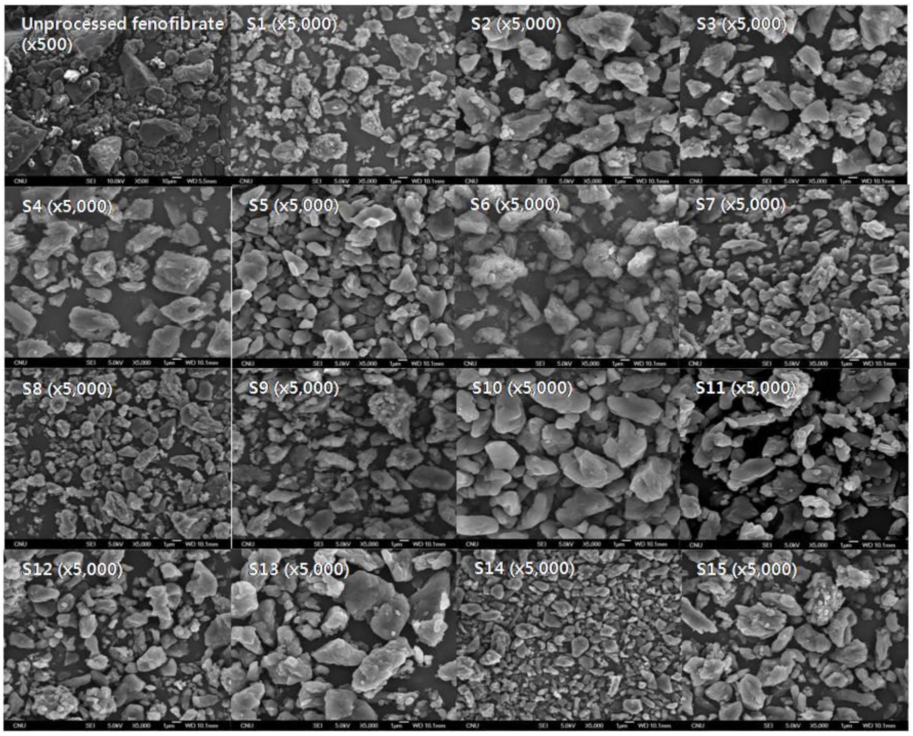

Figure S8. The SEM micrographs of the SA-SD processed fenofibrate/Sucroester 15 microcomposite particles.

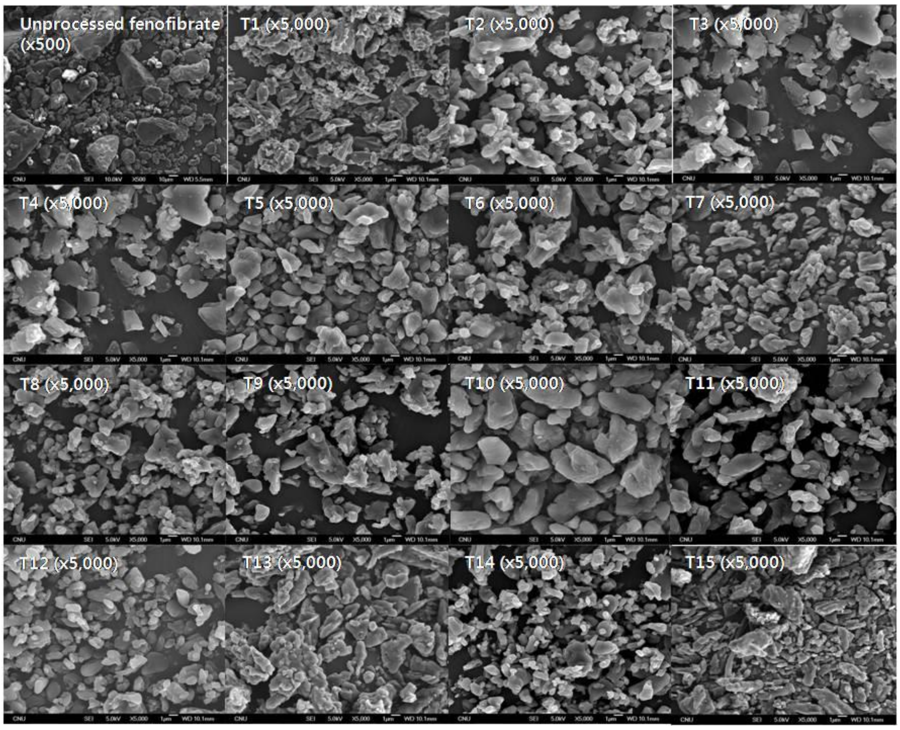

Figure S9. The SEM micrographs of the SA-SD processed fenofibrate/TPGS 15 microcomposite particles.

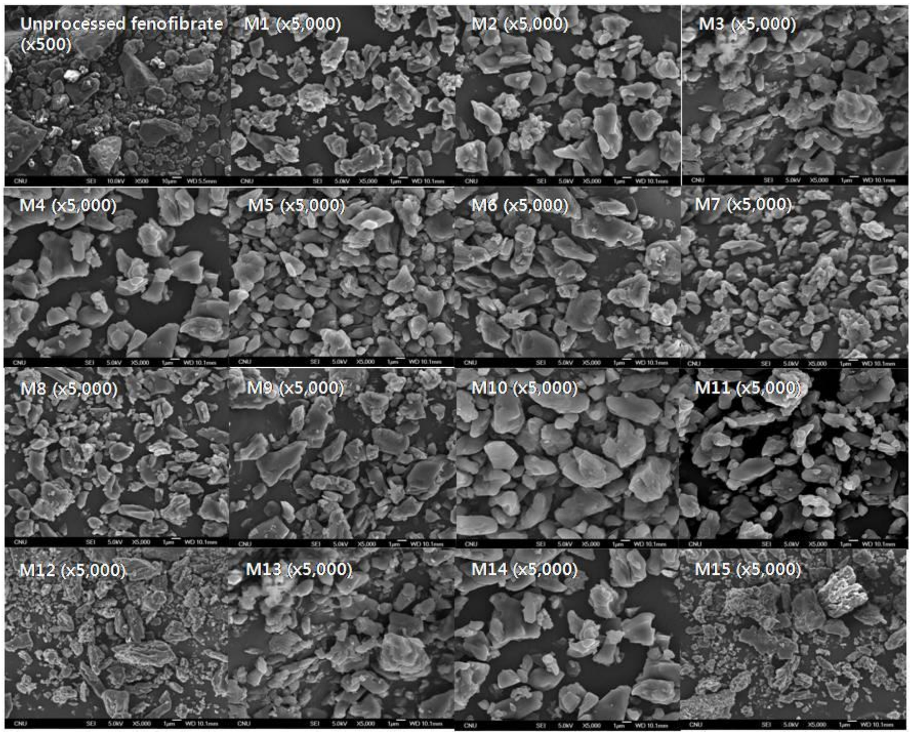

Figure S10. The SEM micrographs of the SA-SD processed fenofibrate/Myrj 52 microcomposite particles.

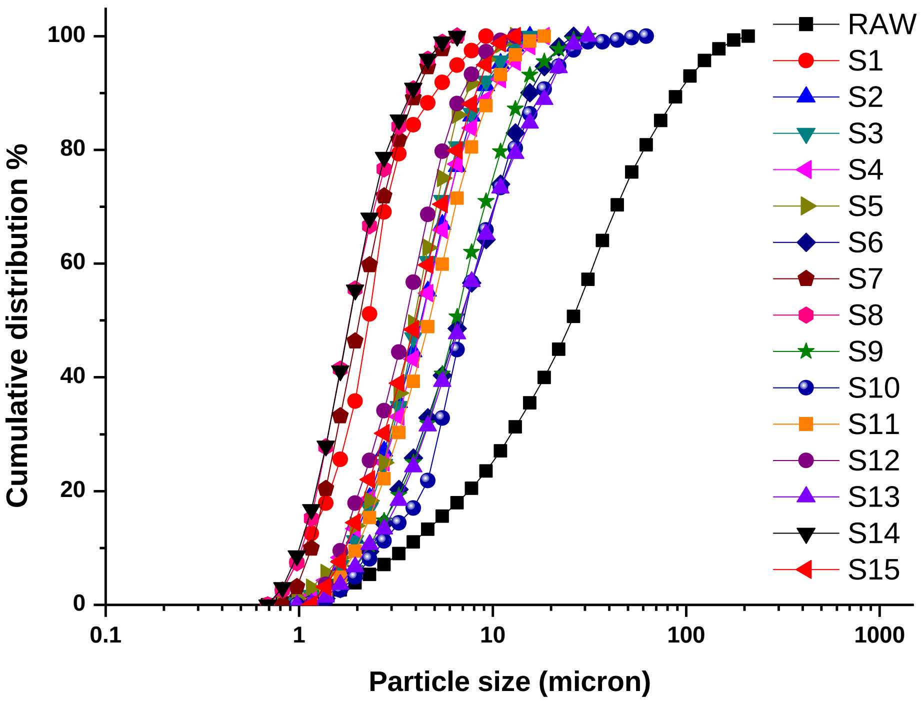

Figure S11. The cumulative particle size distributions of the SA-SD processed fenofibrate/ Sucroester 15 microcomposite particles.

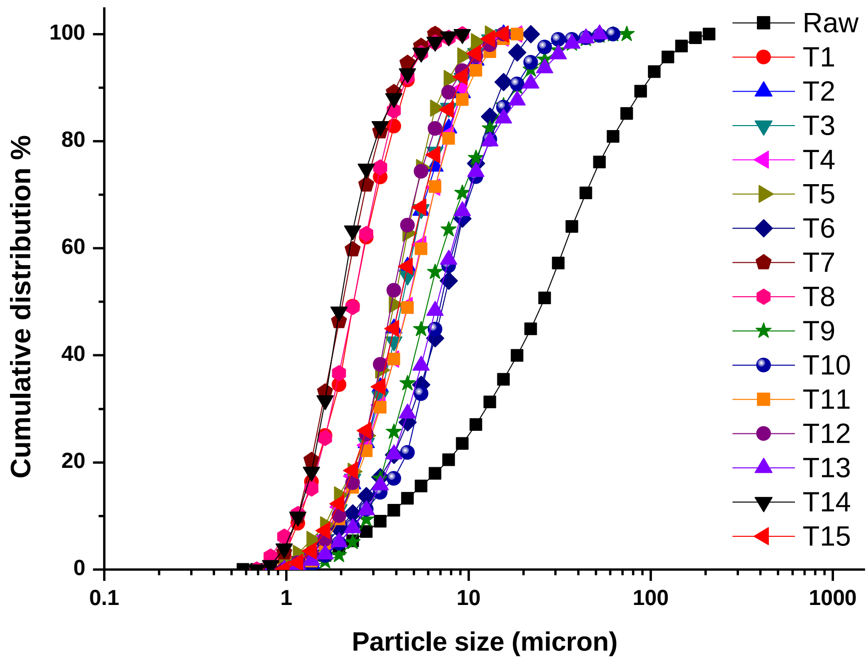

Figure S12. The cumulative particle size distributions of the SA-SD processed fenofibrate/TPGS 15 microcomposite particles.

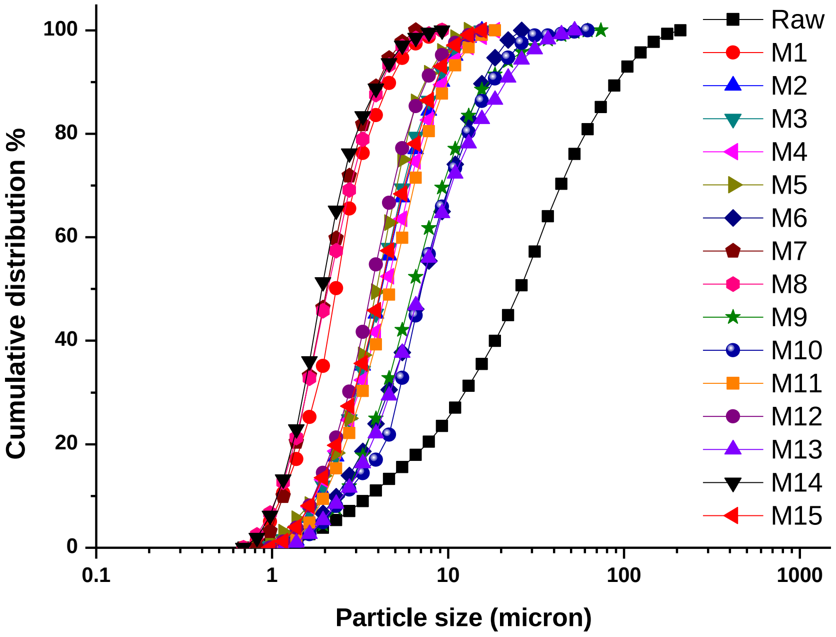

Figure S13. The cumulative particle size distributions of the SA-SD processed fenofibrate/Myrj 52 microcomposite particles.

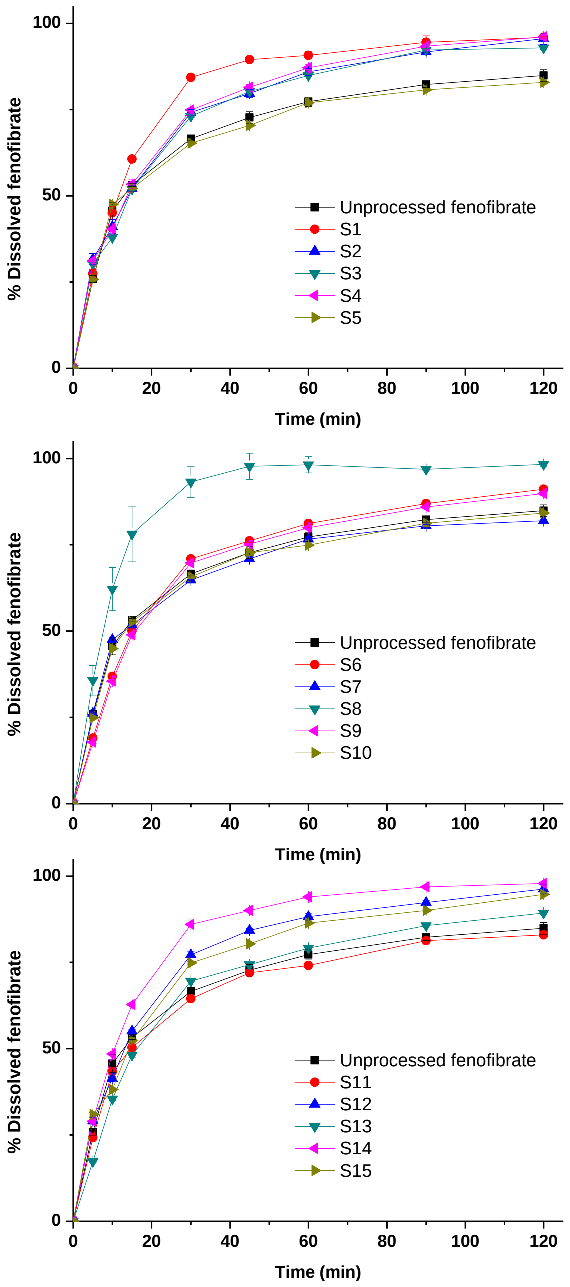

Figure S14. Dissolution profiles of the SA-SD processed fenofibrate-additive microcomposite particles (fenofibrate/Sucro-ester 15).

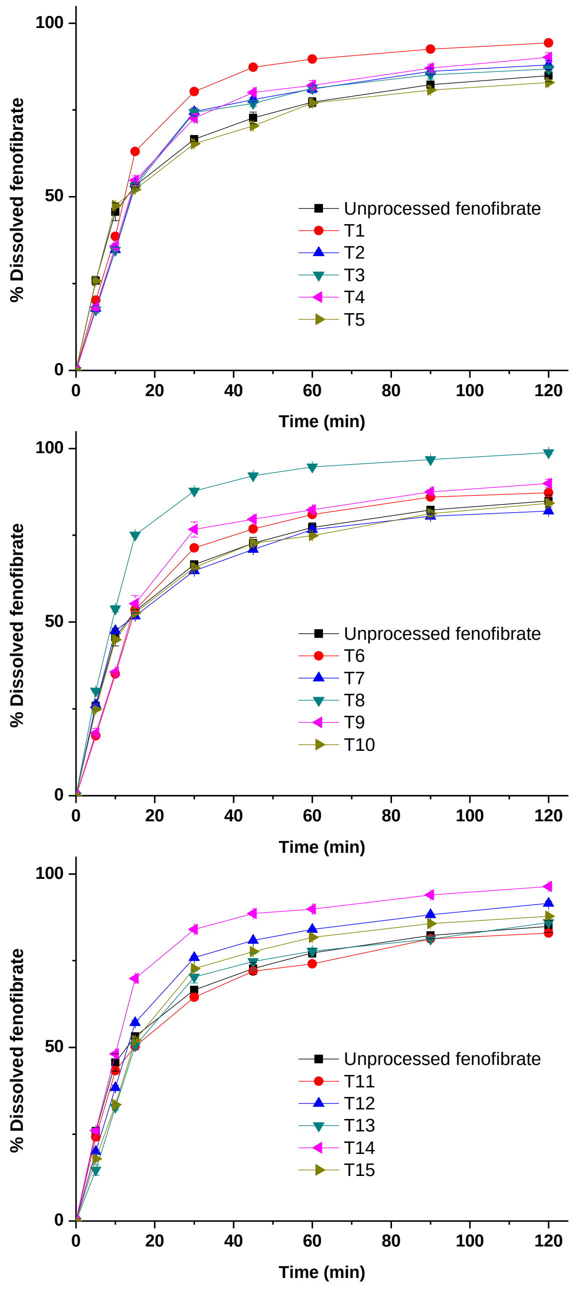

Figure S15. Dissolution profiles of profiles of the SA-SD processed fenofibrate-additive microcomposite particles (fenofibrate/TPGS).

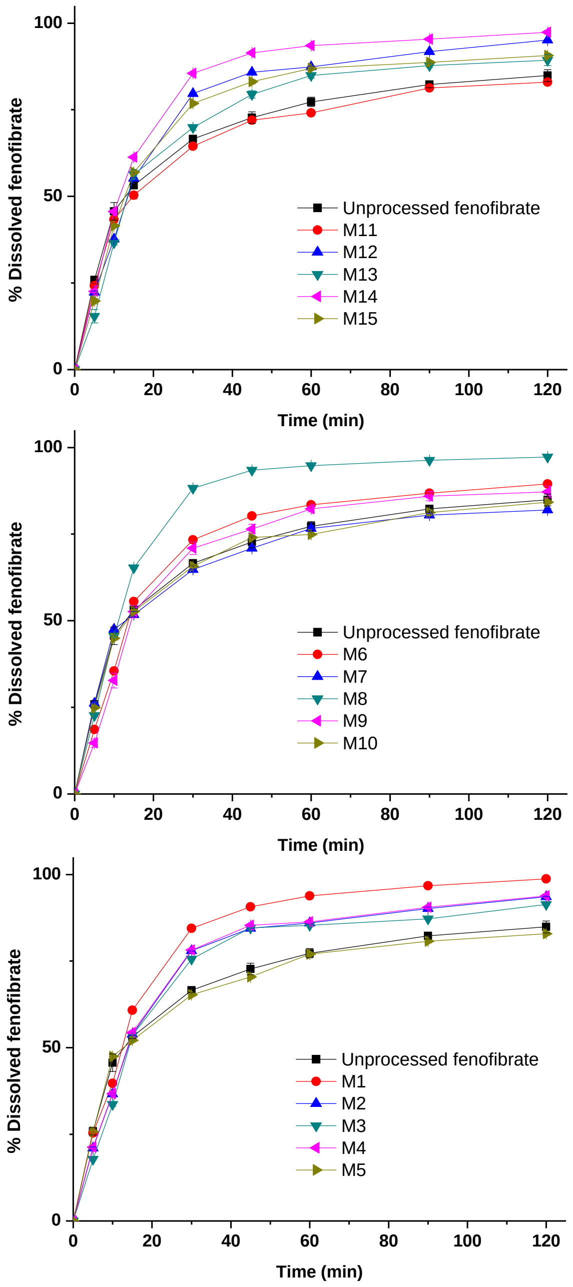

Figure S16. Dissolution profiles of profiles of the SA-SD processed fenofibrate-additive microcomposite particles (fenofibrate/Myrj 52).
